# Resin Glycosides from *Ipomoea funis* as Inhibitors of P‑Glycoprotein in Multidrug-Resistant Breast Carcinoma Cells

**DOI:** 10.1021/acs.jnatprod.5c01273

**Published:** 2026-01-08

**Authors:** Pedro de Jesús Flores-Tafoya, Jennifer Alexis Rojas-Morales, Adriana Carolina Hernández-Rojas, Mabel Fragoso-Serrano, Nohemí Salinas-Jazmín, Elihú Bautista, Martha Lydia Macías-Rubalcava, Rogelio Pereda-Miranda

**Affiliations:** a Departamento de Farmacia, Facultad de Química, Universidad Nacional Autónoma de México, Ciudad Universitaria, Ciudad de México 04510, México; b Secretaría de Ciencia, Humanidades, Tecnología e Innovación and División de Biología Molecular, Instituto Potosino de Investigación Científica y Tecnológica A. C., San Luis Potosí, S.L.P. 78216, México; c Departamento de Farmacología, Facultad de Medicina. 7180 Universidad Nacional Autónoma de México, Mexico 04510, México; d Departamento de Productos Naturales. Instituto de Química, Universidad Nacional Autónoma de México, Ciudad Universitaria, Ciudad de México 04510, México

## Abstract

*Ipomoea funis* Cham. & Schltdl. is an endemic vine found in central Mexico. The use of heart-cutting and peak-shaving methods in recycling preparative HPLC yielded funisin I (**1**), an undescribed resin glycoside, along with the known intrapilosins I (**2**) and V (**3**). Funisin I features operculinic acid A (**6**) as the oligosaccharide core. The structural similarities observed for funisin I align with those previously reported for purginoside I (**4**); however, a difference was apparent in the occurrence of dodecanoic and (−)-(2*R*)-methylbutyric acids as the long- and short-chain fatty acid substituents in compound **1**. Moreover, the structure of the previously described acutacoside F (**5**) was corrected by comparing its NMR data with those of **1** and **4**. The three isolated glycolipids (**1**-**3**) did not show intrinsic cytotoxicity. However, intrapilosin I (**2**), when combined (50 μM) with a sublethal concentration of the antineoplastic drug vinblastine at 0.004 μM, significantly improved its cytotoxic effect and ability to reverse the vinblastine-resistant phenotype in MCF-7 cells by arresting the cell cycle at the G2/M phase and acting as a competitive substrate for P-gp. Resin glycosides could become promising alternatives for developing new therapeutic combinatory strategies to combat multidrug resistance in cancer treatment.

## Graphical abstract

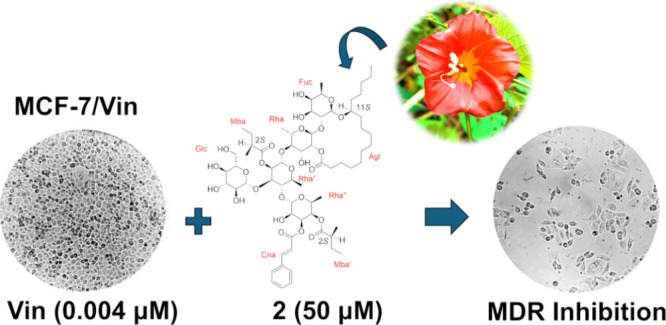

*Ipomoea funis* Cham. Schltdl. is a climbing vine with large, red-orange flowers (Figures S1A and S1B), found exclusively in the mesic environments such as cloud and pine forest borders, damp gullies, and riparian areas in central Mexico.^1^ This plant belongs to the Convolvulaceae, or morning glory family, which includes approximately 2,000 species.^2^ Mexico and Brazil have the highest number of native morning glory species in the world, each with around 60 endemics, including *I. funis*.^1^ In these two megadiverse countries, independent traditional medicinal plant complexes have been established with purgative properties, including members of the genera *Ipomoea* and *Operculina*, which have storage roots, commonly known as jalap.^3^ The most economically significant example of a convolvulaceous root is *I. batatas* (L.) Lam., commonly known as sweet potato, whose use as an edible tuber crop dates to pre-Hispanic times.

*I. funis* has been classified as belonging to the Quamoclit clade (16 spp.), which contains species with significant medicinal value, such as *I. quamoclit* L. and *I. hederifolia* L.^2^ In traditional medicine, the leaves and seeds of *I. quamoclit* have been utilized as a purgative and febrifuge, as well as for the treatment of ulcers, chest pain, carbuncles, and hemorrhoids.^4^ In addition, the hydroalcoholic and hexane extracts of this plant have been shown to exhibit anticancer, antioxidant, antimicrobial, insecticidal, and antidiabetic properties.^4–6^ In contrast, the seeds of *I. hederifolia* are used in traditional Chinese and Indian medicinal practices due to their recognized anti-inflammatory, cathartic, diuretic, and expectorant properties.^7^ Furthermore, this plant material has also demonstrated cytotoxic and anticancer potential, highlighting its medicinal relevance.^8^

The Quamoclit clade represents a significant source of resin glycosides, a distinctive class of secondary metabolites that is exclusive to the Convolvulaceae family.^9,10^ In nature, these glycolipids predominate in their glycosylated macrolactone form, comprising C_14_–C_18_ hydroxy- or dihydroxylated fatty acids, and with up to seven sugar units present in their oligosaccharide structure. Resin glycosides have been demonstrated to induce bowel movements, functioning as potent osmotic laxatives.^3^ They have also been observed to manifest a wide range of biological activities, notably their capacity to modulate the multidrug resistance (MDR) phenotype in cancer cells,^3,11,12^ a phenomenon frequently associated with the overexpression of P-glycoprotein (P-gp).^13^ Recent research has shown the reversal of the MDR phenotype and the induction of cellular chemical sensitization in response to these metabolites when combined with antineoplastic agents, such as vinblastine and podophyllotoxin.^14,15^

*I. funis* is an intriguing subject of investigation due to its close relation to relatives from which biologically active resin glycosides have been isolated: *I. quamoclit*, the source of the quamoclinic acids A-H and quamoclins I–VII,^16^ *I. hederifolia*, which contains hederifolic acids A-D,^17^ and *I.* × *multifida* (Raf.) Shinners, which produces the multifidins I-IX and the multifidinic acids A-G, with cytotoxic activity against human leukemia cells.^18–21^ This study aims to highlight the significance of overlooked convolvulaceous species, as they could lead to the discovery of new bioactive chemical structures, with a particular emphasis on intracellular pathways related to MDR and their potential use in combination cancer therapies.

## Results and Discussion

### Isolation and Structure Elucidation of Funisin I (1)

The total dried extract CH_2_Cl_2_-MeOH (1:1), prepared from *I. funis* was subjected to separation by CC and monitored by TLC, affording a total of six combined fractions (Figure S1E-H), from which fraction 3 (F3) was selected due to its high yield and ^1^H NMR profile, which indicated the presence of resin glycosides (Figure S2). Separation of F3 by reversed-phase preparative HPLC allowed the isolation of three major peaks (Figure S3). Peak 1 (*t*_R_ 9.9 min) did not correspond to a resin glycoside mixture according to its NMR spectra. The purification of peak 2 (*t*_R_ 11.4 min) by recycling HPLC yielded compound **1** (Figure S4), which was further characterized as a novel chemical entity, named as funisin I (**1**) (Figure 1). While the recycling of peak 3 (*t*_R_ 13.2 min) produced two previously isolated glycolipids from *I. intrapilosa* Rose,^22^ the intrapilosins I (**2**) and V (**3**), the latter a structural isomer of compound **1** (Figure 1).

**Figure 1:**
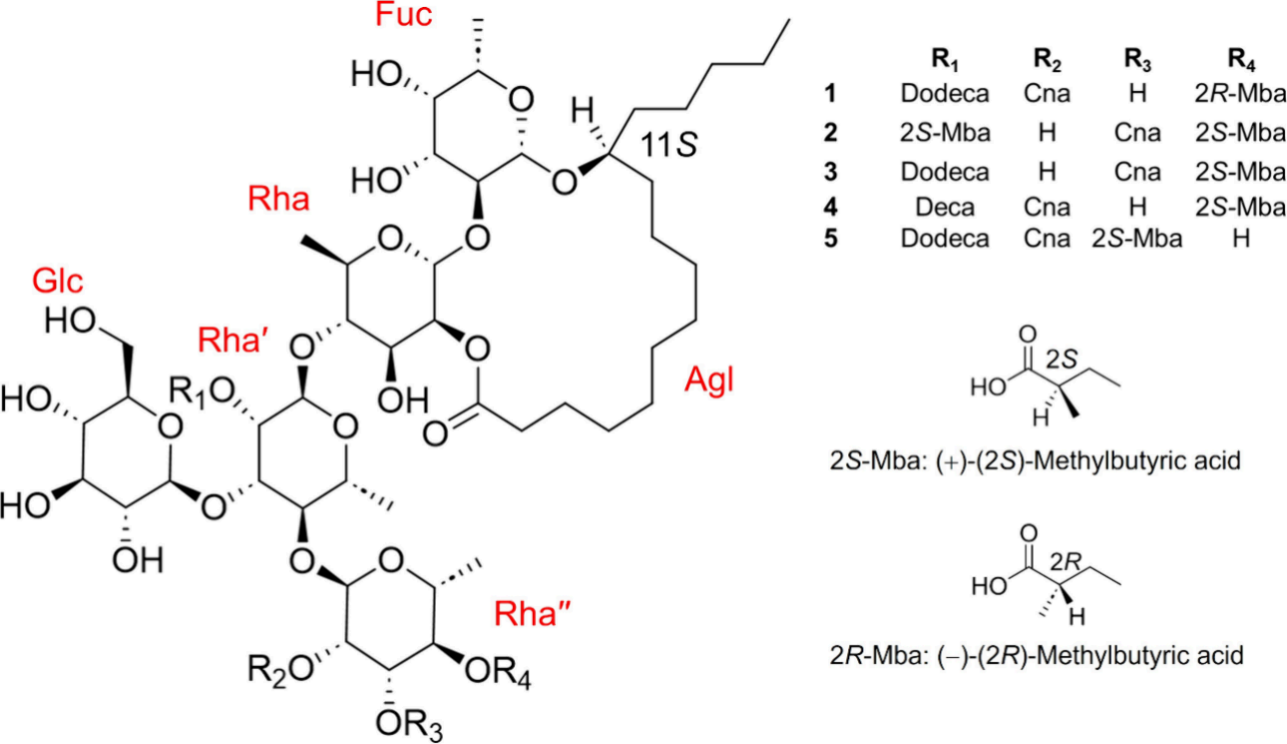
Chemical structures for compounds **1**–**3** and other related glycolipids (**4**–**5**).

The subsequent analysis of the HRESIMS spectra recorded in positive mode for compound **1** allowed the identification of two positively charged ions: the protonated molecule [M + H]^+^ with *m*/*z* 1397.78525 (theoretical value required is *m*/*z* 1397.78276, calcd. error: 1.8 ppm) for the molecular formula [C_72_H_117_O_26_]^+^ and the [M + Na]^+^ adduct with *m*/*z* 1419.77036 (theoretical value required is *m*/*z* 1419.764705, calcd. error: 3.9 ppm) for the molecular formula [C_72_H_116_O_26_Na]^+^ (Figure S5).

After saponification of this natural product, its glycosidic acid core was identified as operculinic acid A (**6**) (Figure S6),^23^ (11*S*)-jalapinolic acid 11-*O*-β-d-glucopyranosyl-(1 → 3)-*O*-[α-L-rhamnopyranosyl-(1 → 4)]-*O*-[α-L-rhamnopyranosyl-(1 → 4)]-*O*-[α-L-rhamnopyranosyl-(1 → 2)]-β-D-fucopyranoside. Comparison of its physical and spectroscopic data (Figure S7) with the previously described lipopentasaccharide core isolated from *Operculina hamiltonii* (G.Don) D.F.Austin & Staples,^14,15^ *I. leptophylla* Torr.,^24^ and *I. purga* Hayne^25^ was made to confirm its chemical structure and to ensure the sequence of glycosylation and the absolute configuration for the major glycosidic acid found in *I. funis.*

The elucidation of the chemical structure of compound **1** was based on the following 1D and 2D NMR experiments: ^1^H (Figure S8), ^13^C (Figure S9), ^1^H–^1^H COSY (Figures S10 and S11), TOCSY (Figure S12), HSQC (Figures S13 and S14), and HMBC (Figures S15). Lactonization and esterification positions as well as the sequence of glycosylation were determined through HMBC analysis (^3^ *J*_CH_): A) to verify the glycosylation sequence, the following connectivities were observed (Figure S16): Fuc C-1/Agl H-11 (δ_C_ 104.6, δ_H_ 3.87), Rha C-1/Fuc H-2 (δ_C_ 98.9, δ_H_ 4.18), Rha C-4/Rha′ H-1 (δ_C_ 81.5, δ_H_ 5.94), Rha′ C-3/Glc H-1 (δ_C_ 80.3, δ_H_ 5.10), Rha′ C-4/Rha″ H-1 (δ_C_: 78.6, δ_H_ 6.34); B) to establish the sites of esterification (Figure S17): Dodeca C-1/Rha′ H-2 (δ_C_ 174.1, δ_H_ 6.30), Cna C-1/Rha″ H-2 (δ_C_ 167.2, δ_H_ 6.31), Mba C-1/Rha″ H-4 (δ_C_ 176.8, δ_H_ 5.83); and C) for the site of lactonization (Figure S17): Agl C-1/Rha H-2 (δ_C_ 173.4, δ_H_ 5.94). The diversity of esterifying groups found in compound **1** is comparable to that of purginoside I (**4**) from *I. purga*, as they are both derived from operculinic acid A (**6**) (Figure S6), and share the same pattern of substitution, differing only in the presence of dodecanoic acid in the case of **1**, as the long-chain fatty acid substituent, replaced with decanoic acid in **4** which was used as a structural model for further NMR comparison^25^ (Figure S18). Finally, the absolute configuration of the chiral ester, methylbutyric acid (Mba), was determined to be *R*, based on its registered levorotatory optical value of [α]^22^_589_ – 10.0, as previously described for intrapilosin IV.^22^ All these analyses allowed for the identification of the natural product funisin I (**1**) as the new: 11­(*S*)-hydroxyhexadecanoic acid 11-*O*-β-d-glucopyranosyl-(1→3)-*O*-[α-L-rhamnopyranosyl-(1→4)-*O*-(2-*O*-*n*-dodecanoyl)]-α-L-rhamnopyranosyl-(1→4)-*O*-[α-L-rhamnopyranosyl-(1→2)*-O*-(2-*O*-cinnamoyl)-(4-*O*-(2*R*)-methylbutanoyl)]-β-D-fucopyranoside-(1, 2-lactone). Table 1 condenses the assigned chemical shift values of the ^1^H and ^13^C nuclei for this compound.

**Table 1:** NMR Spectroscopic Data (700 MHz, pyridine-*d*_5_) of Funisin I (**1**)­^a^

position	**δ**_**C**_ **, type**	**δ**_**H**_ **(** *J* **in Hz)**
**Fuc-1**	104.6, CH	4.75 d (7.4)
**2**	78.6, CH	4.18 dd (9.1, 7.4)
**3**	74.1, CH	4.08 dd (9.1, 3.8)
**4**	73.2, CH	3.99 brs
**5**	71.1, CH	3.77 q (6.4)
**6**	17.7, CH_3_	1.53 d (6.4)
**Rha-1**	98.9, CH	5.52 s
**2**	73.7, CH	5.94 brs (2.8, 1.9)
**3**	69.7, CH	5.04 dd (9.4, 2.8)
**4**	81.5, CH	4.17 dd (9.4, 9.4)
**5**	69.2, CH	4.49 dq (9.4, 6.7)
**6**	19.4, CH_3_	1.66 d (7.0)
**Rha′-1**	100.1, CH	5.94 brs
**2**	73.9, CH	6.30 dd (3.0, 1.5)
**3**	80.3, CH	4.81 m
**4**	78.6, CH	4.44 dd (9.4, 9.4)*
**5**	68.7, CH	4.49 dq (9.4, 6.0)
**6**	18.3, CH_3_	1.55 d (6.0)
**Rha″-1**	100.1, CH	6.34 brs
**2**	73.2, CH	6.31 dd (3.0, 2.0)
**3**	68.5, CH	4.81 m
**4**	75.0, CH	5.83 t (9.6)
**5**	68.7, CH	4.48 dq (9.6, 6.4)
**6**	19.5, CH_3_	1.70 d (6.4)
**Glc-1**	105.3, CH	5.10 d (7.8)
**2**	74.9, CH	3.99 t (9.6)
**3**	80.3, CH	4.15 m*
**4**	71.6, CH	3.98 t (9.6)
**5**	78.2, CH	3.83 ddd (9.0, 5.5, 2.0)
**6a**	63.3, CH_2_	4.13 m
**6b**	63.3, CH_2_	4.44 m
**Agl-1**	173.4, C	
**2a**	34.8, CH	2.33 ddd
**2b**	34.8, CH	2.49 ddd
**11**	82.6, CH	3.87 m
**16**	14.6, CH_3_	0.89 t (6.9)
**Dodeca-1**	174.1, C	
**2**	34.6, CH_2_	2.37 t (7.5)
**12**	14.6, CH_3_	0.88 t (7.3)
**2-mba-1**	176.8, C	
**2**	41.8, CH	2.55 m
**2-Me**	17.3, CH_3_	1.24 d (7.1)
**3**	27.4, CH_2_	1.79 m
**4**	11.9, CH_3_	0.93 t (7.4)
**Cna-1**	167.2, C	
**2**	118.9, CH	6.38 d^16^
**3**	145.5, CH	7.68 d^16^

a Chemical shifts and coupling constants are expressed in ppm and hertz, respectively. *Signals overlapping.

### Structure Correction of Acutacoside F (**5**)

A previous study proposed the same substitution pattern for **1** to an isolated resin glycoside from *Argyreia obtusifolia* Lour. (syn. *Argyreia acuta* Lour., Convolvulaceae),^26^ named as acutacoside F (**5**). However, for this suggested structure, the previously literature data did not align with those registered for our isolated glycolipid, such as the physical properties (melting point and optical rotation) and spectroscopic data (^1^H and ^13^C NMR). A thorough examination of the ^1^H NMR spectrum of **5** led us to conclude that C-4 in Rha″ is attached to a free – OH group, based on the H-4 signal being upfield-shifted at δ_H_ 4.09 (Figure S19). If C-4 were esterified as proposed, a downfield shilft (ca. δ + 1.5 ppm) would be expected, as seen in the operculins from *Operculina macrocarpa*. In operculin XIII, which has a free −OH group, a doublet of doublets (seen as a triplet-like signal) is observed at δ_H_ 4.26 (*J =* 9.0, 9.0 Hz). In contrast, in operculin XV, this C-4 Rha″ signal is centered at δ_H_ 5.77 (*J =* 9.0, 9.0 Hz) because of the acylation by dodecanoic acid.^27^ In acutacoside F, no correlation was found between this signal and the carbonyl of the (2*S*)-Mba in the HMBC experiment. Instead, the interaction of the latter with the proton in the Rha″-3 position at δ_H_ 6.00 (*J* = 10.0, 3.1 Hz) was observed, which appeared as a clear doublet of doublets (Figure S20).

The analysis of ^13^C and HSQC confirmed the structure of funisin I (**1**) by comparison of its anomeric region with that of acutacoside F (**5**).^26^ A substantial difference was found between these two anomeric regions. In funisin I (**1**), only four signals were observed at δ_C_ 98.9, 100.1, 104.6, and 105.4, which was due to the overlapping of the Rha′-1 and Rha″-1 signals, instead of the five distinct signals seen at δ_C_ 98.6, 99.3, 100.3, 104.6, and 105.6, corresponding to Fuc-1, Rha-1, Rha′-1, Rha″-1 and Glc-1 in **5**. It is noteworthy that this overlapped pattern of anomeric chemical shifts was also previously observed in purginoside I (**4**), as previously reported by us,^25^ where the Rha′-1 and Rha″-1 signals at δ_C_ 100.0 provided additional evidence for the substitution pattern in **1** (Figure 2). This observation was corroborated by the interactions found between these overlapped ^13^C signals and its corresponding geminal ^1^H resonances in HSQC, where two correlations for the same chemical shift were spotted at δ_H_ 5.94 and 6.34. Despite being derived from operculinic acid B, acutacoside D exhibits ^13^C similarities in this region to purginoside I and funisin I (Figure 2), as Rha′-1 and Rha″-1 are nearly superimposed at δ_C_ 99.7 and 99.8,^28^ respectively.

**Figure 2:**
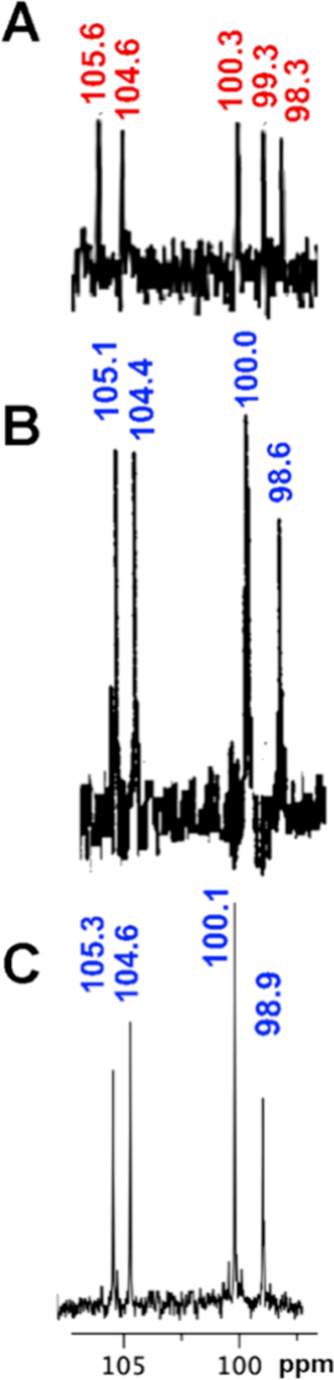
^13^C NMR spectra in pyridine-*d*_5_ for the anomeric signals of related pentasaccharides: (A) acutacoside F (**5**). Adapted spectrum portion with permission from the open access article distributed by a CCBY license by MDPI; see, *Molecules* 2017, 22(3), 440; 10.3390/molecules22030440; (B) purginoside I (**4**); (C) funisin I (**1**).

Finally, the position of lactonization at Rha-2 in compound **1** was determined by comparison of the chemical shifts of the diastereotopic protons in α position to the carbonyl in the macrolactone ring (Figure S21). These α-CH_2_ protons in **1** are placed at δ_H_ 2.33 and 2.49, resembling those of purginoside I (δ_H_ 2.26 and 2.44), and other resin glycosides; for example, operculins XIII (δ_H_ 2.25 and 2.44), XIV (δ_H_ 2.27 and 2.46),^27^ and hamiltonin I (δ_H_ 2.26 and 2.34),^14^ all of which have their lactonization at Rha-2. This differs significantly in cases where the ring is closed at Rha-3, like in operculin XII (δ_H_ 2.22 and 2.68) and batatinoside IX (δ_H_ 2.28 and 2.67).^27,29^ Considering all the above, the former structure of acutacoside F (**5**) was corrected as 11­(*S*)-hydroxyhexadecanoic acid 11-*O*-β-d-glucopyranosyl-(1→3)-*O*-[α-L-rhamnopyranosyl-(1→4)-*O*-(2-*O*-*n*-dodecanoyl)]-α-L-rhamnopyranosyl-(1→4)-*O*-[α-L-rhamnopyranosyl-(1→2)*-O*-(2-*O*-cinnamoyl)-(3-*O*-(2*S*)-methylbutanoyl)]-β-D-fucopyranoside-(1,2-lactone). It is important to mention that such structural reassignment also represents a novel chemical entity in the literature.

Among the 25 categories of glycosidic acids with a pentasaccharide core, operculinic acid A is regarded as the most common type. To date, approximately 70 glycolipids derived from operculinic acid A have been isolated.^9,10,30^ These acyl sugars most commonly incorporate residues of 2(±)-methylbutyric, *n*-decanoic, *n*-dodecanoic, and *trans*-cinnamic acids as acylating groups of the glycosidic core at positions Rha′-2, Rha″-2, and Rha′′-4. The latter can be explained by the enzymatic activity of acyltransferases, which act with high promiscuity as ester donors, exhibiting regioselectivity for the esterification position at the acyl acceptor moiety in the Convolvulaceae and Solanaceae species.^31,32^

### Intramolecular Transesterification

Although initially believed to be a new resin glycoside, compound **3** was shown to be a known isomer of funisin I (intrapilosin V). Both isomers coexist in the collected peak 2, as confirmed by HPLC. Overloading the column with a high concentration of the collected peak 2 (a mixture of **1** and **3**) enabled sufficient accumulation of the minor isomeric compound **3**, via purification by recycling semipreparative HPLC.^33^ After purification, acetylation of the collected peak with *t*_R_ 275 min, produced peracetylated versions of funisin I (**7**) and intrapilosin V (**8**) (Figure S22), according to their spectroscopic data derived from 1D and 2D NMR experiments (Tables S1 and S2). This spectroscopic analysis confirmed the occurrence of an intramolecular transesterification for the *trans*-cinnamic unit between positions C-2 and C-3 of the terminal rhamnose unit in funisin I (**1**) to form intrapilosin V (**3**). Peracetylation halted the reaction and produced a mixture of isomeric products **7** and **8** at equilibrium, in a 2:1 ratio. Noticeable differences in the aforementioned positions were determined by ^1^H NMR as follows (Figure S23): Rha″-2 (δ_H_ 6.15 for **7**, 5.47 for 8) and Rha″-3 (δ_H_ 5.96 for **7**, 6.04 for **8**). HMBC determined the sites of esterification in each compound by contrast with those recorded for **1** (Figures S24 and S25). Such a reaction was catalyzed by pyridine-*d*_5_ at room temperature during the recording of the NMR spectra.^34,35^ A mechanism of reaction for this 1,2-intramolecular nucleophilic acyl migration was proposed (Figure S26) based on former descriptions for other resin glycosides, namely, pescapreins X-XVII from *I. pes-caprae* (L.) R.Br.^36^ and evolvulins II and III from *Evolvulus alsinoides* (L.).^37^ In all these cases, both hydroxy groups must be on the same side (*cis* or *syn*) relative to the axis of the chair conformation of the sugar unit involved for this transesterification to occur. Such a requirement is present in funisin I (**1**) and intrapilosin V (**3**), allowing the formation of the 5-membered cyclic *ortho*-ester transition state, which is key to the migration of the acylating residue (Figure S26).

### Cytotoxicity Evaluation

The cytotoxicity of compounds **1**-**3** was assessed by the sulforhodamine B (SRB) assay^38^ against both parental (MCF-7) and MDR (MCF-7/Vin) breast carcinoma cells. None of the compounds exhibited intrinsic cytotoxicity (IC_50_ > 50 μM) in contrast to the inhibitory activity of vinblastine (MCF-7 IC_50_ 0.04 μM; MCF-7/Vin IC_50_ 0.4 μM) and podophyllotoxin (MCF-7 IC_50_ 0.03 μM; MCF-7/Vin IC_50_ 0.4 μM) (Figure S27 and Table S3), allowing for further evaluation of their inhibitory effects in combination with a sublethal concentration of these control drugs (0.004 μM). Table 2 summarizes the half-maximal inhibitory concentration (IC_50_) values for each compound in combination with the two antineoplastic drugs, reflecting their ability to inhibit the growth of cancer cells (Figure 3). Among all these resin glycosides, compound **2** showed the best inhibitory effects in both MCF-7 cell lines. When combined at 20 μM with 0.004 μM vinblastine, compound **2** was able to reduce MCF-7/Vin cell viability by 34% (IC_50_ 31.9 μM, Figure 4), and by 22% when combined with podophyllotoxin (IC_50_ 34.7 μM, Figure 4). Similar potentiating effects have been observed in MCF-7/Vin cells treated with other noncytotoxic glycolipids.^11,12,15^

**Figure 3:**
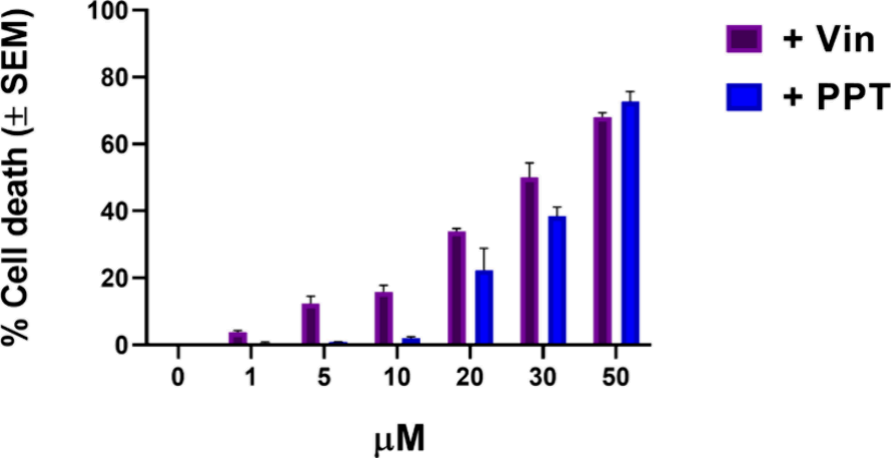
Viability of MCF-7/Vin cells after 72 h of combination therapy using the SRB assay: cells were exposed to concentrations (1, 5, 10, 20, 30, and 50 μM) of intrapilosin I (**2**) and a subinhibitory concentration of vinblastine and podophyllotoxin (0.004 μM). Each experiment was performed three times independently (*n* = 3). Values are expressed as the percentage of the control and represent means ± SEM. Abbreviations: Vin, vinblastine; PPT, podophyllotoxin.

**Table 2:** Half-Maximal Inhibitory Concentration (IC_50_ μM) of Different Combinations of Compounds **1**–**3** with a Sublethal Concentration of Antineoplastic Drugs

	IC_50_ (μM)							
	1			2			3	
sample^a^	MCF-7	MCF 7/Vin		MCF-7	MCF 7/Vin		MCF-7	MCF-7/Vin
Vin	18.3 ± 1.5	36.8 ± 1.5		3.76 ± 2.5	31.9 ± 0.33		18.17 ± 3.77	34.46 ± 5.34
PPT	36.1 ± 1.4	40.3 ± 1.3		12.87 ± 0.97	34.7. ± 1.9		32.82 ± 1.2	49.3 ± 1.48

a Compounds **1**–**3** were tested at 1, 5, 10, 20, 30, and 50 μM to enhance cytotoxic effects of antineoplastic agents: vinblastine and podophyllotoxin (parental and MDR type: 0.004 μM). Each experiment was performed three times independently (*n* = 3). Abbreviations: Vin, vinblastine; PPT, podophyllotoxin. Values are expressed as the percentage of the control and represent means ± SEM.

**Figure 4:**
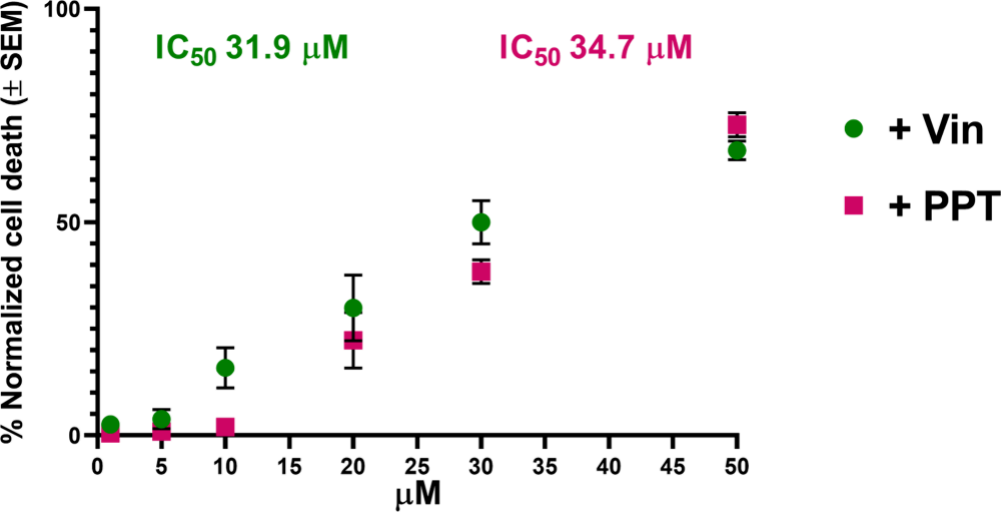
Concentration–response curves for MCF-7/Vin cells incubated with increasing concentrations of intrapilosin I (**2**) and a subinhibitory concentration of 0.004 μM of vinblastine and podophyllotoxin. Values are expressed as the percentage of the control and represent means ± SEM. Each experiment was done in triplicate (*n* = 3).

Although structure–activity relationships (SAR) were not established for these glycolipids, it is noteworthy that, as with other cytotoxic and efflux pump-inhibitory glycolipids, their amphiphilic nature governs the interactions with their biological targets.^12,39^ In resin glycosides, amphiphilicity is driven by structural features that play a crucial role in their overall biological outcome, such as the presence or absence of a macrolactone ring, free – OH groups on specific sugar units, and the type and substitution pattern of acyl groups in the sugar core.^10^ SAR studies of ipomoeassin F (from *I. squamosa* Choisy) analogues have highlighted the importance of some structural traits, emphasizing the presence of the α, β-unsaturated esters, particularly the cinnamate moiety,^40^ suggesting that unsaturated fatty acids may covalently bind resin glycosides to their molecular cellular targets, such as the pore-forming subunit α of the isoform 1 of the protein transport Sec61 at the endoplasmic reticulum membrane.^41^ Other important structural features are the macrocyclic skeleton and the natural 11*S* configuration in the macrolactone,^41,42^ which have been associated with cytotoxic effects comparable to those of paclitaxel, a commonly used drug in cancer therapy. Such traits are present in compounds **1**-**3** and could not only help explain the cytotoxic responses observed for these glycolipids but also support the idea that this class of compounds, like in the case of **2**, can act as cell-cycle inhibitors.^42^ Such mechanisms of action have been described for ipomoeassin F, which arrests the G1 phase, accompanied by a decrease in the number of mouse fibroblasts in the G2/M interphase,^43^ and for aquaterin IV (*I. aquatica* Forssk.), which constrains proliferation of HepG2 cells in the G0/G1 interphase followed by apoptosis.^42^

Light microscopy analysis of cell viability revealed that intrapilosin I (**2**, 50 μM) exhibited low cytotoxicity in both parental MCF-7, and vinblastine-resistant (MCF-7/Vin) human breast carcinoma cells compared to the control group. However, when combining **2** with a sublethal concentration of vinblastine (0.004 μM), a significant decrease in the survival of both phenotypes was observed, suggesting a synergistic effect (Figure 5). Both MCF-7 and MCF-7/Vin cells without treatment maintained their characteristic polygonal morphology even after a 72-h prolonged incubation. On the contrary, significant morphological changes were observed in the presence of compound **2** and its combination with vinblastine in both phenotypes, such as suspended cells with damaged membranes and apoptotic bodies. Similar alterations in morphology, such as chromatin condensation and fragmentation (chromatinorrhexis), have been observed in HepG2 cells after their exposure to aquaterin II.^44^ Such an effect is similar to that of vinblastine alone at higher concentrations (Figure S28). This is consistent with previous descriptions of glycolipids, such as farbitins A-F from *I. nil* (L.) Roth, syn. *Pharbitis nil* (L.) Choisy.,^45^ which enhanced vincristine activity in KB/VCR cells, and hamiltonins I–IV from *O. hamiltonii*, which increased vinblastine cytotoxicity in MCF-7/Vin cells.^14,15^

**Figure 5:**
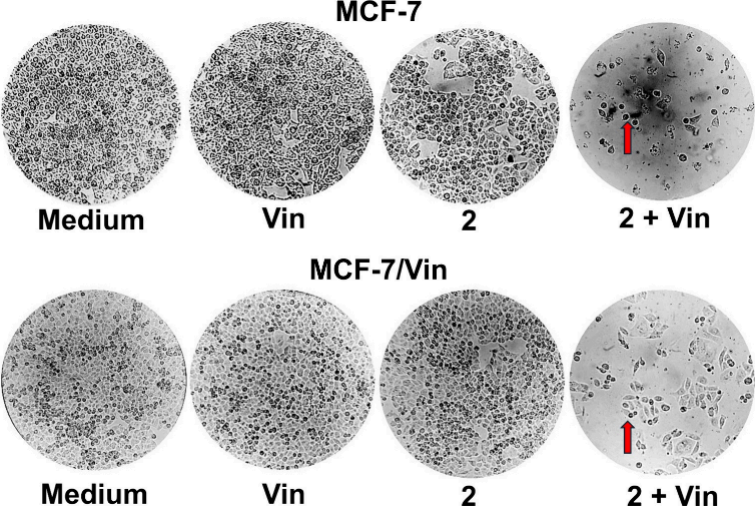
Chemical sensitization in breast carcinoma cells by intrapilosin I (**2**) in combination with vinblastine. Representative images of vinblastine-sensitive MCF-7 cells and vinblastine-resistant MCF-7/Vin cells after 72 h of treatment with vinblastine (Vin, 0.004 μM), intrapilosin I (**2**, 50 μM), or their combination (**2** + Vin) using optical microscopy. Breast carcinoma cells in adherence, growing in medium and independent treatments of Vin and **2**, presented their characteristic shapes, while morphological changes in suspended cells with apoptotic bodies (red arrows) were observed in the combination therapy assay (**2** + Vin).

### Cellular Death Induction by Intrapilosin I (**2**)

To investigate the mechanism of cellular death induced by intrapilosin I (**2**), annexin V/7-AAD assays were performed. These confirmed that the combination of **2** with vinblastine significantly increased late apoptotic and necrotic populations in MCF-7 cells but not in MCF-7/Vin cells (Figure 6A). In MCF-7, apoptosis correlated with caspase-3 activation, as evidenced by reduced levels of procaspase-3 and increased cleaved caspase-3 on Western blot (Figure 6B). In contrast, the caspase-3 cleavage was not observed in MCF-7/Vin cells after treatment. Additional assays using different treatments (Figure S29) confirmed the lack of apoptotic response in the resistant phenotype, indicating that compound **2** in combination with vinblastine does not trigger caspase-dependent apoptosis in MCF-7/Vin cells. These data agree with descriptions correlating resin glycosides and the mitochondrial apoptotic pathway.^42^ However, the absence of this effect in MCF-7/Vin suggests that compound **2** might modulate alternative mechanisms in multidrug-resistant (MDR) cells.

**Figure 6:**
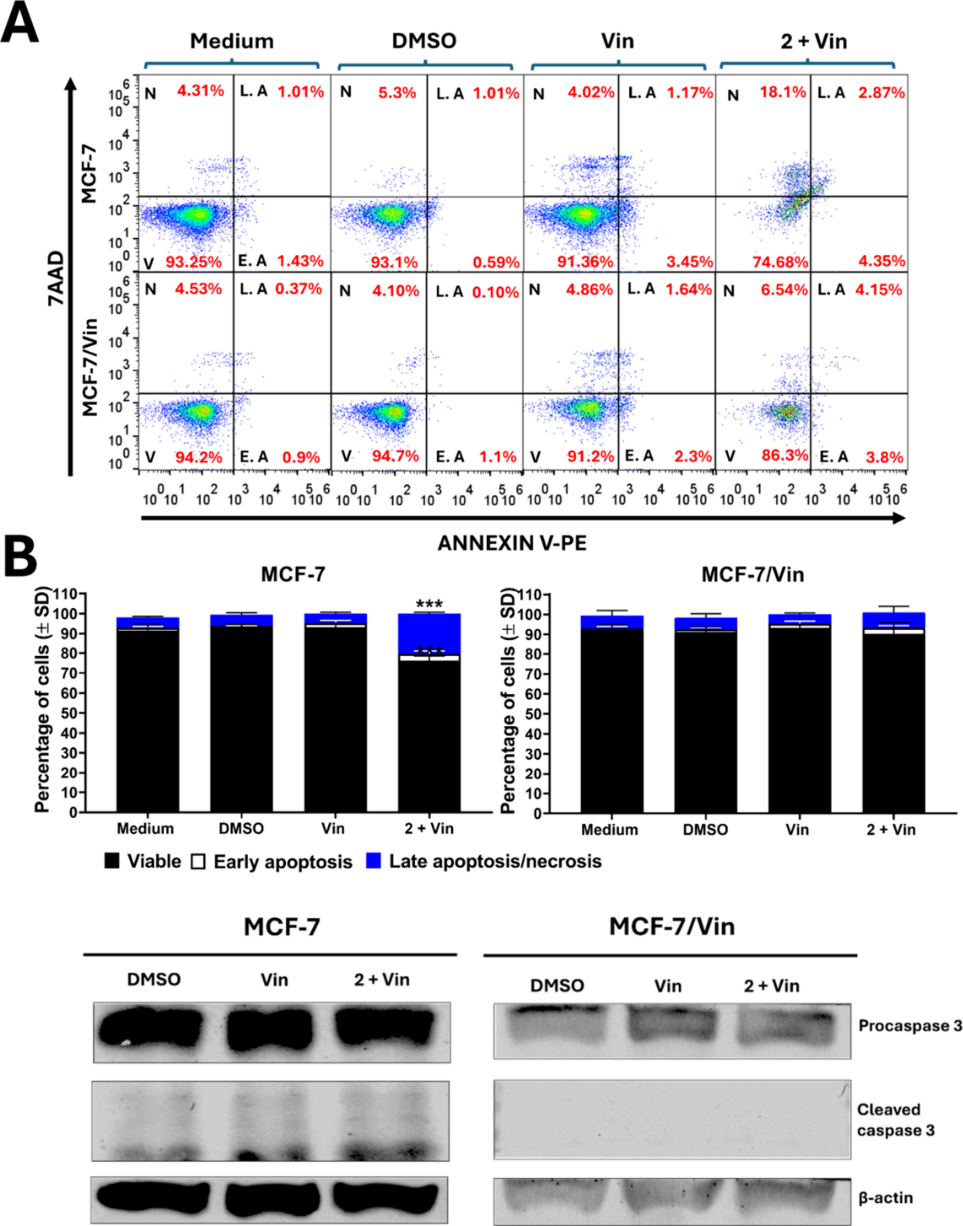
Effect of intrapilosin I (**2**) on apoptosis: (A) flow cytometry analysis with double staining using annexin V/PE and 7AAD in MCF-7 and MCF-7/Vin cells, bars represent the percentage of early and late apoptosis in the different cell phenotypes, data are presented as mean ± SD from three independent experiments. *** *p* < 0.01; (B) Western blot analysis of apoptosis-associated proteins (caspase-3) in MCF-7 and MCF-7/Vin cells after the indicated treatments for 48 h. Abbreviations: N, necrosis; LA, late apoptosis; EA, early apoptosis; V, viable.

Cell-cycle analysis by propidium iodide (PI) staining revealed that intrapilosin I (**2**) induced a G2/M interphase arrest in both cell lines, accompanied by a decrease in G0/G1 and S phases (Figures 7A and 7B). This effect was exacerbated in MCF-7/Vin when treated with compound **2** and vinblastine, suggesting that this glycolipid sensitizes resistant cells to the antiproliferative chemotherapy. Similar effects have been observed with other resin glycosides such as aquaterins, which caused G0/G1 arrest mediated by cyclin-dependent kinase inhibition, a mechanism that could explain the observed results.^42^

**Figure 7:**
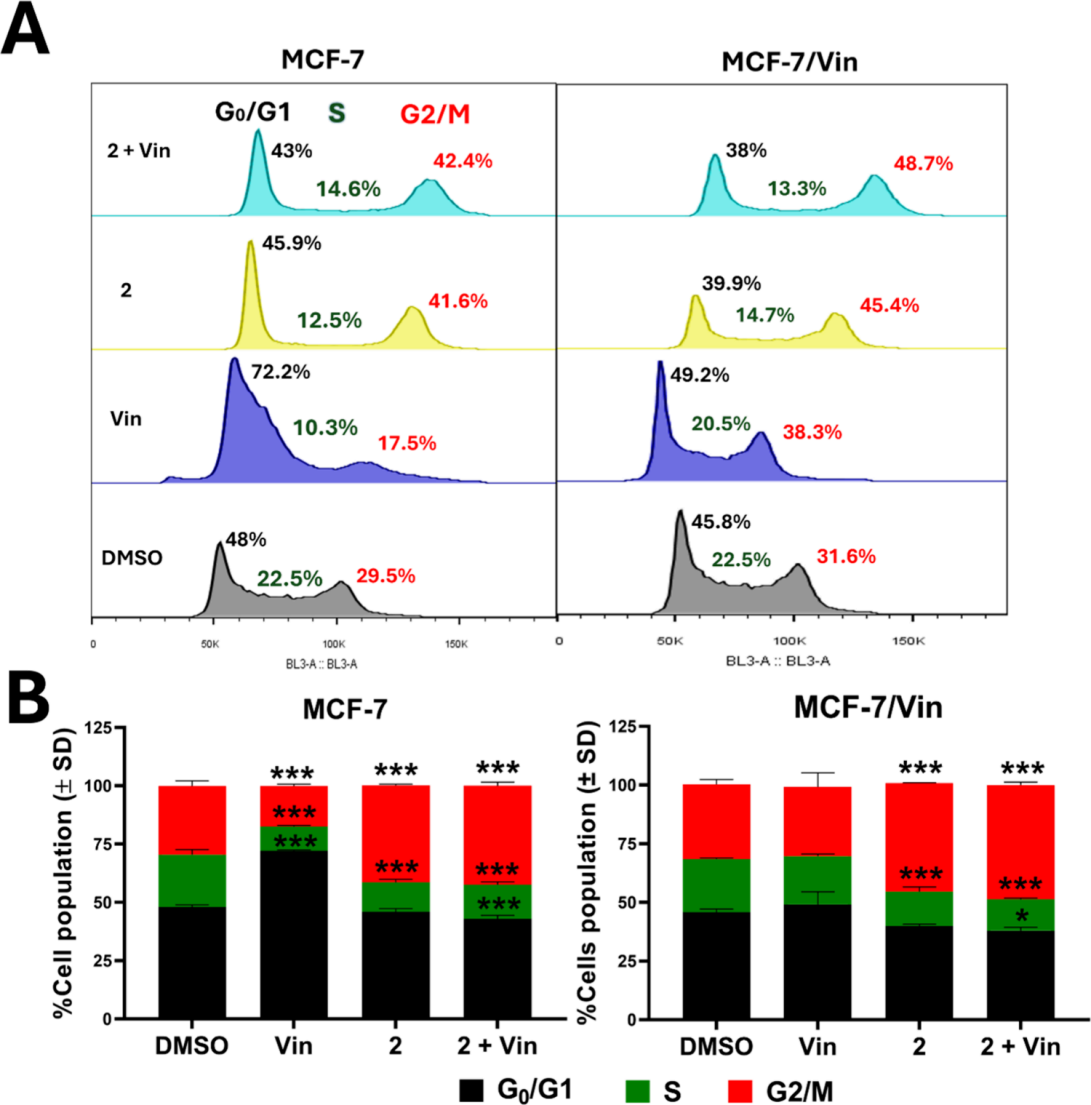
Effect of intrapilosin I (**2**) on the MCF-7 cell cycle. (A) Graphical representation of cell cycle distribution in parental (MCF-7) and vinblastine-resistant (MCF-7/Vin) cells following treatment with vinblastine (Vin, 0.004 μM), intrapilosin I (**2**, 50 μM), or their combination (**2** + Vin). (B) Quantification of cells in distinct cell cycle phases post-treatment. Results, expressed as percentages, represent the mean ± SD of three independent experiments (*p < 0.05, ***p < 0.001).

### Synergistic Sensitization of Drug-Resistant Cells

The association of the MDR phenotype with ABC efflux pumps in both MCF-7 and MCF-7/Vin cells was determined by Western blot (Figure 8). P-glycoprotein (P-gp) was overexpressed in MCF-7/Vin cells compared to parental MCF-7 cells, confirming its role in drug resistance in this cell line, consistent with previous descriptions.^46^ Notably, treatment with compound **2** in combination with vinblastine reduced P-gp levels, suggesting a synergistic effect that is capable of effectively sensitizing P-gp-dependent MDR cells.

**Figure 8:**
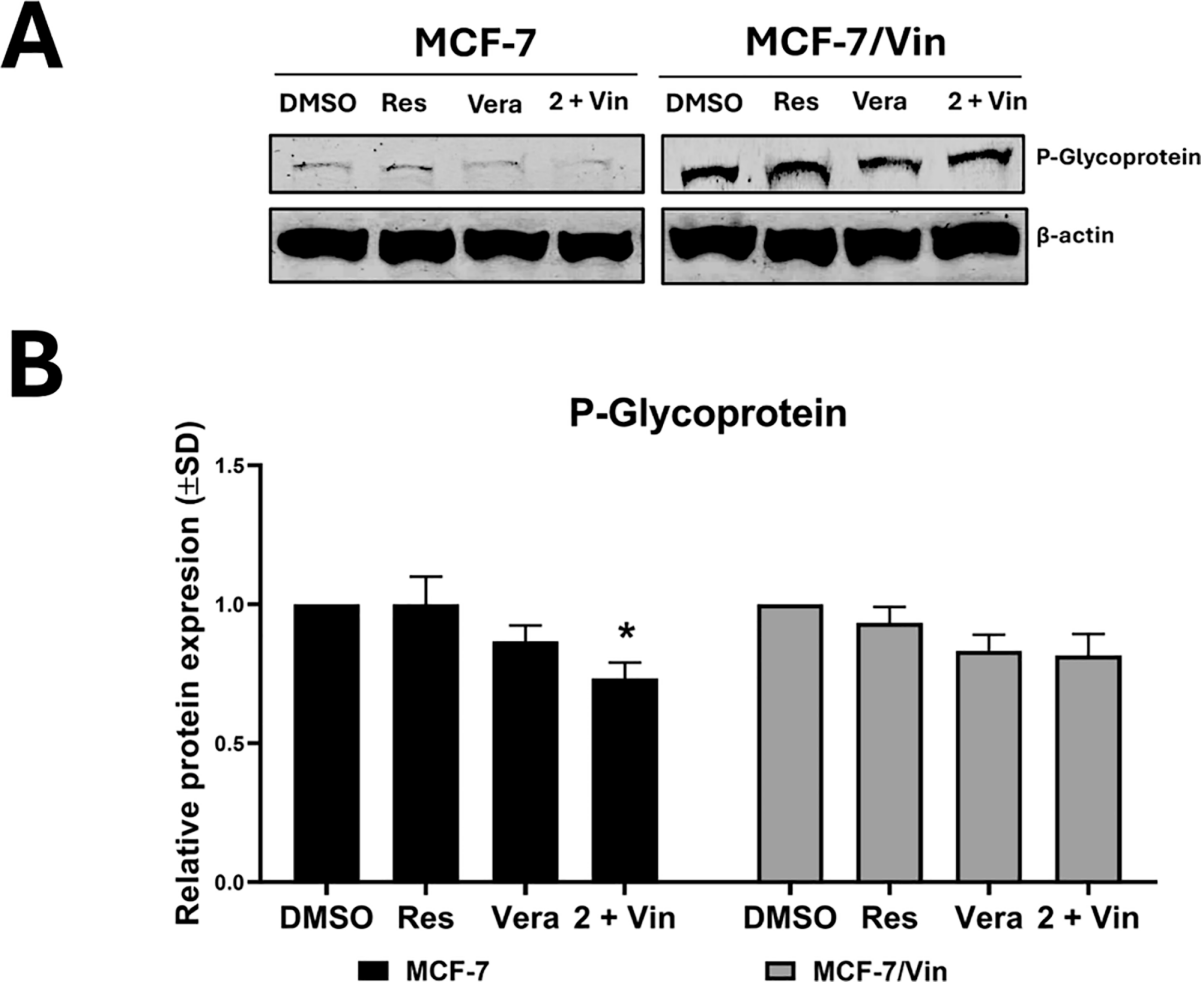
Effect of intrapilosin I (**2**) on proteins of ABC efflux pumps. (A) Western blot analysis of protein associated with ABC efflux pump (P-glycoprotein) in MCF-7, and MCF-7/Vin (vinblastine-resistant) cells following the indicated treatments for 48 h, and (B) relative densitometric quantification of the analyzed protein bands normalized to β-actin. Data are presented as mean ± SD (*n* = 3), *p < 0.05, **p < 0.005, ***p < 0.001.

The impact of intrapilosin I (**2**) on the transport of rhodamine 123 (Rho123), a recognized fluorescent substrate of P-gp,^11^ was investigated. As shown in Figure 9, in the absence of treatment, MCF-7/Vin cells cleared most of Rho123 after 30 min of incubation, contrasting with parental cells, which confirms that vinblastine resistance in the cells involves efflux pumping associated with P-gp overexpression. However, the addition of compound **2** in combination with vinblastine partially inhibited Rho123 efflux, showing intracellular accumulation comparable to that induced by reserpine and verapamil (Figure 10). These results explain the increased sensitization of drug-resistant MFC-7/Vin cells (Figure 6). This finding suggested that compound **2** could act as a competitive substrate for P-gp, promoting the intracellular accumulation of chemotherapeutic agents. This mechanism has been described for other natural compounds, such as glycosylated flavonoids, selected terpenoids and saponins and the resin glycosides purgin II (*I. purga*) and murucoidin V (*I.murucoides*), which exert dual effects by interfering in both the expression and function of P-gp, possibly due to their amphiphilic structures that facilitate interactions with the transporter transmembrane domains.^47–50^ Therefore, the observed potentiation effect by noncytotoxic glycolipids, may result from enhanced drug internalization, leading to an increase in intracellular drug concentration and improved efficacy of cytotoxic drugs.

**Figure 9:**
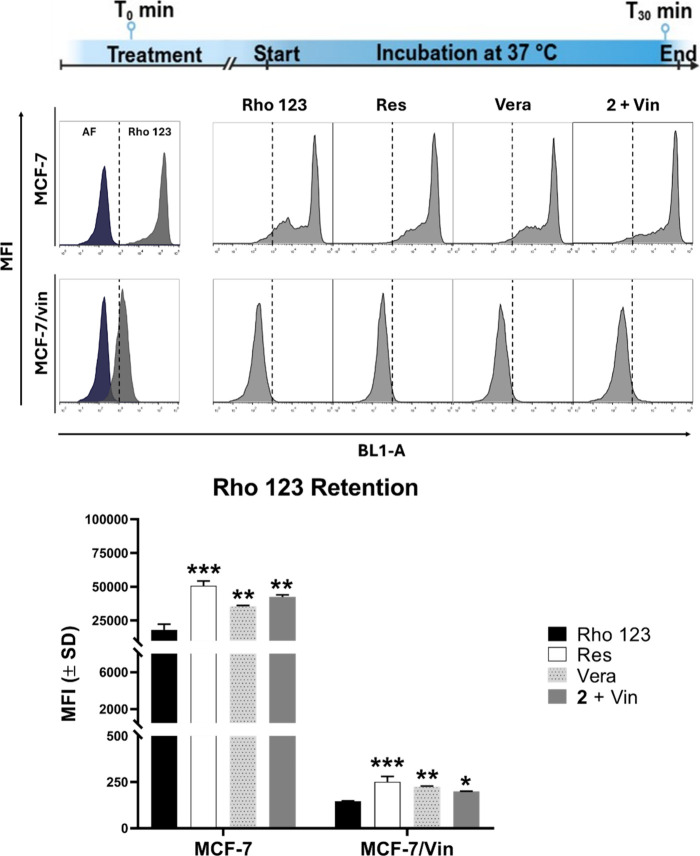
Effect of intrapilosin I (**2**) on ABC efflux pumps activity. Flow cytometry analysis of rhodamine 123 (Rho123) retention in MCF-7 and MCF-7/Vin cells. Cells were incubated for 30 min at 37 °C with reserpine (Res, 10 μM), verapamil (Vera, 10 μM), and intrapilosin I with vinblastine (C2 + Vin, 50 + 0.004 μM) in the presence of Rho123. The fluorescence of cells incubated without the fluorescent probe (AF) was used as a control. Results represent the mean ± SD of three independent experiments (*p < 0.05, **p < 0.005, ***p < 0.001).

## Conclusions

The present study sets a remarkable precedent for future investigations of other convolvulaceous species native to tropical regions that have been neglected over the years. It focuses on *I. funis*, an endemic Mexican plant, with no previous records of its chemical composition and biological potential. The isolation of funisin I (**1**), an undescribed lipopentasaccharide derived from operculinic acid A, shows that the native Mexican species from the Quamoclit clade could be a source for discovering new bioactive molecules. Furthermore, it could function as an extension to the knowledge of additional sources for known related resin glycosides, such as the intrapilosins. The biological results described for intrapilosin I (**2**) make significant contributions to the understanding of the mechanism of action of resin glycosides as efflux-pump and cell-cycle inhibitors. Furthermore, these findings recognize the potential therapeutic repurposing of well-known bioactive molecules, which have been more deeply investigated in recent years as repositioned anticancer therapeutics regulating several signaling pathways which include the inhibition of P-gp and inducing programmed cell death in various types of cancer cells.^51^ In a similar manner, new approaches could incorporate approved antineoplastic drugs like vinblastine or podophyllotoxin in combination with molecules such as resin glycosides to address the multidrug resistance phenomenon in cancer therapy.

## Experimental Section

### General Experimental Procedures

Melting points were acquired uncorrected on a Fisher-Johns 220 VAC apparatus (Thermo Scientific). Optical rotation values were obtained on a PerkinElmer model 341 polarimeter using MeOH as solvent. UV measurements were recorded on a PerkinElmer model 365 UV/vis spectrophotometer inside a quartz cell. IR measurements were performed on a PerkinElmer model Spectrum 400 FTIR/FIR spectrophotometer equipped with an ATR accessory with a resolution of 4 cm^–1^, total number of scans: 64, and a spectral range: 4000–400 cm^–1^. NMR spectra were recorded on a Bruker AVANCE III HD (700 MHz) spectrometer on Norelltubes (3 mm × 178 mm) containing pyridine-*d*_*5*_ (sample volume: 600 μL) and tetramethylsilane was used as an internal standard. Positive-ion HRESIMS data were acquired on a Waters Xevo G2 XS UPLC-ESI-QTof system. MS conditions: capillary: 2.0 kV; cone: 15 V; ion source temperature: 150 °C; interface temperature: 350 °C; scan speed, 2 scans s^–1^; mass range: 300–2200 amu; solvents: MeCN/MeOH/formic acid (0.1%). GC-MS analysis was conducted on a PerkinElmer GC Clarus SQ 8C spectrometer under the following GC conditions: capillary column VF-5 ms (30 × 0.25 mm, film thickness 0.25 μm); He linear velocity: 30 cm/s; 50 °C isothermal for 4 min, linear gradient to 300 at 40 °C/min; and MS conditions: ionization energy: 70 eV; ion source temperature: 250 °C; interface temperature: 250 °C; mass range 35–550 amu. Column chromatography (CC) was carried out on silica gel 60–200 mesh (Merck). Thin-layer chromatography (TLC) was performed on aluminum plates (25 × 50 mm) impregnated with silica gel coated with fluorescent indicator F254 (Merck). Plate reading was done under UV light at 254 and 365 nm and with an acidified cerium sulfate solution. Reversed-phase HPLC analysis was conducted on equipment adapted with a recycling pump (Waters 600) and coupled to a refractive index detector (Waters 2414). HPLC data were analyzed with Waters Empower2 software.

### Plant Material

The examined material included the complete fresh vines, leaves, and scarce flowers and seeds (17 kg) collected on May 17, 2023, by A.C. Hernández-Rojas and M. Kilian. This plant grows in the locality of Cinco Palos, Municipality of Coatepec, State of Veracruz, Mexico (Lat: 19.499446 N Long: – 96.969251 W, elevation 1400 m asl). The tropical cloud forest of this locality is a special formation of shallow soils over limestone rock outcrops. Here, *I. funis* is a resilient species, rarely developed as an enormous woody vine with around 5 cm of diameter, in a border of the forest in a community constituted by *Quercus* spp., *Piper* spp., *Ctenitis melanosticta* (Kunze) Copel., *Peperomia chazaroi* G.Mathieu & T.Krömer, *Telanthophora grandifolia*, (Less.) H.Rob. & Brettell, *Cnidosculus*, *Hoffmannia*, *Clusia*, *Phanerophlebia*, *Bomarea*, and abundant epiphytes of the genus *Tillandsia*. Duplicates of the species were identified by Dr. Hernández-Rojas at the XAL Herbarium (accession numbers: 0155065, 0156262, 0156263, 0156264, and 156265) from Instituto de Ecología A.C. (INECOL, Xalapa, Veracruz, Mexico), and by Dr. Travis Marsico at the STAR Herbarium (Arkansas State University Campus Jonesboro, Arkansas, USA), identified as STAR037613 and STAR037614 (Figure S1C).

### Extraction and Isolation

To determine the presence of resin glycosides, an initial TLC profiling of a preliminary extract (CH_2_Cl_2_–MeOH, 1:1) was conducted by comparison with the known glycolipid tricolorin A from *I. tricolor* Cav.^40^ This analysis allowed to conclude that the extract contained such class of compounds. Consequently, the total ground plant material (3.5 kg) was subjected to maceration with the aforementioned solvent mixture. The direct-dried product (194.2 g), a greenish brown-amber resinous residue (Figure S1D), yielded six combined fractions after column chromatography (CC) in Si gel (Figure S1E-H). Fraction 3 (F3, CH_2_Cl_2_–MeOH, 7:3) was selected for further chemical analysis due to its high yield (23.6 g) and TLC-NMR profile, which showed coloration similarities (yellowish brown spotting on plate) to tricolorin A^40^ and a high complexity for sugar signals in ^1^H NMR (Figure S2). The application of the heart-cutting technique by refractive-index recycling preparative HPLC of F3 with a μBondapak amino (125 Å, 10 μm, 19 × 150 mm) column and CH_3_CN-MeOH (9:1), as the mobile phase (flow rate: 5 mL/min), allowed the separation of three major peaks with retention times as follows: peak 1 (*t*_R_ 9.9 min), peak 2 (*t*_R_ 11.4 min), and peak 3 (*t*_R_ 13.2 min) (Figure S3). Recycling HPLC of peak 2 under the previously described analytical conditions afforded lipopentasaccharide **1** (14.9 mg) while peak 3 yielded the known intrapilosins I (**2**; 20.0 mg) and V (**3**; 25.6 mg). In addition, a complex mixture of two structural isomers was also identified by NMR after peracetylation (Ac_2_O-pyridine, 2:1) of a supplementary amount of compound **3** isolated during the purification process of **1** (Figure S4).

*Funisin I (* **1** *)*: white solid; ORD (*c* 1.9 MeOH) [α]_589_ – 14.2, [α]_578_ – 15.3, [α]_546_ – 17.4, [α]_436_ – 28.4, [α]_365_ – 40.5; UV (MeOH) **λ**_**max**_ (log ε) 280 (0.75) nm (Figure S30); FTIR (ν_**max**_): 3422, 3064, 2926, 2855, 1720, 1637, 1515 1451, 1378, 1277, 1136, 1071 cm^–1^ (Figure S31); ^1^H (700 MHz, pyridine-*d*_*5*_; Figure S8) and ^13^C (175 MHz, pyridine-*d*_*5*_; Figure S9) NMR spectroscopic data, see Table 1. HRESIMS *m*/*z* 1397.78525 [M + H]^+^ (calcd. for C_72_H_117_O_26_^+^ requires 1397.78276, δ = 1.8 ppm); *m*/*z* 1419.77036 [M + Na]^+^ (calcd. for C_72_H_116_O_26_Na^+^ requires 1419.764705, δ = 3.9 ppm) (Figure S5).

*Intrapilosin I (* **2** *)*: white solid; ORD (c 0.27 MeOH) [α]_589_ – 18.9, [α]_578_ – 21.1, [α]_546_ – 22.2, [α]_436_ – 35.6, [α]_365_ – 50.0; ^1^H (700 MHz, pyridine-*d*_5_; Figure S32) and ^13^C (175 MHz, pyridine-*d*_5_; Figure S32) NMR spectroscopic data. The purity and identity of this compound was assessed by comparison with an authentic sample of its physical constants and NMR spectroscopic data,^22^ as well as by HPLC coelution experiments (*t*_R_ 11.3 min): μBondapak amino (125 Å, 10 μm, 3.9 × 300 mm) column and CH_3_CN-MeOH (9:1), as the mobile phase (flow rate: 0.4 mL/min).

*Intrapilosin V (* **3** *)*: white solid; ORD (*c* 0.12 MeOH) [α]_589_ – 15.8, [α]_578_ – 16.7, [α]_546_ – 19.2, [α]_436_ – 29.2, [α]_365_ – 44.2; ^1^H (700 MHz, pyridine-*d*_*5*_; Figure S33) and ^13^C (175 MHz, pyridine-*d*_*5*_; Figure S33) NMR spectroscopic data. The purity and identity of this compound was assessed by comparison with an authentic sample of its physical constants and NMR data,^22^ as well as by HPLC coelution experiments (*t*_R_ 19.4 min): μBondapak amino (125 Å, 10 μm, 3.9 × 300 mm) column and CH_3_CN (100%), as the mobile phase (flow rate: 0.4 mL/min).

### Alkaline Hydrolysis of Resin Glycosides

A resin glycoside sample was mixed with a 5% KOH aqueous solution in a reflux setup at 95 °C for a period of 4 h under constant stirring. The reaction solution was later adjusted to pH 4.0 with 1 N HCl and submitted to a series of extractions using Et_2_O and *n*-BuOH (3 × 5 mL). The resulting *n*-BuOH phase was finally washed with deionized water (3 × 5 mL) and dried over anhydrous Na_2_SO_4_ and concentrated under reduced pressure. The basic hydrolysis of **1** liberated operculinic acid A (**6**; Figure S14) and the acylating esters. Direct insertion by GC-MS of the resulting organic phase allowed the identification of 2-methylbutyric acid (Mba, *t*_R_ 4.8 min): *m*/*z* [M]^+•^ 102 (1), 87 (35), 74 (100), 54 (29), 41 (36); *trans*-cinnamic acid (Cna, *t*_R_ 8.1 min): *m*/*z* [M]^+•^ 148 (59), 147 (100), 131 (23), 103 (57), 77 (50), 51 (31), and *n-*dodecanoic acid (Dodeca, *t*_R_ 8.5 min) *m*/*z* [M]^+•^ 200 (12), 171 (10), 157 (26), 129 (27), 115 (18), 85 (32), 73 (100), 60 (59), 43 (69), 41 (74).

*Operculinic acid A (* **6** *):* white solid; mp: 170–172 °C; ORD (*c* 0.17, MeOH) [α]^22^_589_ – 56.5, [α]_578_ – 58.2, [α]_546_ – 64.7, [α]_436_ – 105.9, [α]_365_ – 159.4; For NMR spectroscopic data, see Table S4 and Figure S7. All data were consistent for this glycosidic acid when compared to previous descriptions.^24^

### Absolute Configuration for the Chiral Ester

To determine the absolute configuration of the liberated chiral ester, a portion of the resulting Et_2_O residue from saponification was treated with triethylamine (two drops) and 4-bromobenzyl bromide (10 mg) in dry acetone (5 mL) under stirring for 2 h at room temperature. The product was dried and resuspended in deionized water (5 mL), followed by extraction with Et_2_O (15 mL). Subsequently, the three benzylated derivatives were purified through a process of further evaporation and analysis by normal-phase HPLC, in accordance with a previously reported method of transesterification.^52^ Optical rotation dispersion values for the 4-bromobenzyl 2-methylbutyrate derivative from **1** were as follows: [α]^22^_589_ – 10.0, [α]_578_ – 10.0, [α]_546_ – 13.3 (*c* 1.0, CHCl_3_). Comparison with (*R*)-(−)-benzyl 2-methylbutyrate present in intrapilosin IV, [α]_598_ – 9, [α]_578_ – 9, [α]_546_ – 10.5, (*c* 0.8, CHCl_3_), and (*S*)-(+)-benzyl 2-methylbutyrate from intrapilosin V, [α]_598_ + 9.3, [α]_578_ + 9.6, [α]_546_ + 10.9 (*c* 1.0, CHCl_3_), led to the conclusion that 2-methylbutyric acid in **1** had an *R* configuration.^22^

### Cytotoxicity Evaluation

The American Type Culture Collection (ATCC) cell lines of breast cancer cells (MCF-7), parental and multidrug-resistant (MCF-7/Vin) were incubated in RPMI 1640 medium (31800–022, Gibco) supplemented with 10% FBS (A15–301, Bioevolution), containing 100 U/mL of penicillin G and 100 μg/mL of streptomycin (15140122, Gibco) in a humidified environment at 37 °C. MDR in MCF-7 cells was achieved by continuous exposition to vinblastine with an increasing concentration up to 0.2 μg/mL over a five-year period of time.^11^ Cells were cultivated in their logarithmic growth phase and treated with different sample concentrations (1–50 μM) in triplicate under the aforementioned conditions. A 20% TCA cold solution (70 μL) was added followed by incubation at 4 °C for 30 min. Plates were then washed with running water, dried, and stained with 0.4% sulforhodamine B (SRB, S1402–5MG, Sigma-Aldrich) for another 30 min. A 1% acetic acid solution was used to remove excess SRB succeeded by TRIS buffer addition.^39^ Absorption was determined on an ELISA plate reader (Bio-Tex Instruments) at 545 nm. Vinblastine (V1377–10MG, Sigma-Aldrich) was used as a positive control.

### Synergistic Effect Evaluation

Antineoplastic drugs, namely, vinblastine and podophyllotoxin (P4405–50MG, Sigma-Aldrich) at 0.004 μM were tested in combination with **1**-**3** at different concentrations (1, 5, 10, 20, 30, and 50 μM) using the SRB assay against MCF-7 and MCF-7/Vin. Cells were seeded in 96-well plates at a density of 5 × 10^3^ cells per well for 72 h in a humidified atmosphere with 5% CO_2_. Half-maximal inhibitory concentrations (IC_50_) were calculated by plotting percentage of cell viability over concentration on Prisma v.8.01 software.^14^

### Apoptosis Evaluation

The effect of compound **2** on cell death was evaluated using a double staining assay with the PE Annexin V kit (130–119–353, Miltenyi Biotec) and 7AAD (A9400, Sigma-Aldrich). MCF-7 and MCF-7/Vin cells were seeded in 48-well plates (10^3^ cells/well) in 500 μL of vinblastine-enriched medium (0.004 μM) with compound **2** at 50 μM and incubated at 37 °C for 72 h in humidified atmosphere with 5% CO_2_. After incubation, cells were harvested using trypsin-EDTA (0.05%-1X, In vitro), neutralized with growth medium and centrifuged (200 × *g*/5 min) to afford a cellular pellet, which was further resuspended in Annexin V binding buffer (99.5 μL) and mixed with PE Annexin V (0.5 μL) and 7AAD (5 μL) in FACS tubes. Samples were incubated in the dark at room temperature for 25 min. Finally, cells were analyzed on a flow cytometer (Attune NxT), acquiring a total of 10,000 events per sample.

### Cell Cycle Assay

Cell cycle analysis was performed using propidium iodide (PI) staining (IP P4170, Sigma-Aldrich) according to the manufacturer’s instructions. Cells were seeded in 48-well plates at 10^3^ cells/well for 72 h in a humidified atmosphere with 5% CO_2_. Cells were treated with a sublethal concentration of vinblastine (0.004 μM), and **2** (50 μM). After incubation, cells were harvested using trypsin-EDTA, neutralized with growth medium and centrifuged (200 × *g*/5 min) to afford a cellular pellet, which was further resuspended in PI 500 μL (20 μg/mL diluted in 0.1% NP40) in FACS tubes in the dark at room temperature for 30 min. Samples were analyzed by flow cytometry (Attune NxT) with a total acquisition of 100,000 events.

### Rhodamine 123 Efflux Evaluation

MCF-7 and MCF-7/Vin cells were placed on 48-well plates (9 × 10^3^ cells/well) in culture medium (500 μL) and incubated at 37 °C for 24 h in a humid atmosphere with 5% CO_2_. The ability of cells to retain Rho123 was determined after reaching a 70% confluence. Cells were exposed to compound **2** (50 μM) and Rho123 (40 μM, 83702–10MG, Sigma-Aldrich) for 30 min with fresh medium. At the end of the incubation time, the accumulation of Rho123 was stopped by washing the cells three times with ice-cold PBS. To measure the accumulation of Rho123 in basal state, control cells with probes were measured at time 0 and at 30 min and the mean fluorescence intensity (MFI) was measured by flow cytometry with a total acquisition of 10,000 events. P-gp inhibitor agents, reserpine (10 μM, 7551–5G, Merck) and verapamil (10 μM, R1131–1G, Sigma-Aldrich) were used as positive controls.^11^

### Western Blot Analysis

The reversal of MDR mediated by P-gp and the mechanism of cellular death was evaluated using the following monoclonal antibodies against: caspase-3 (sc-56053, Santa Cruz Biotechnology), ABCB1 (sc-55510, Santa Cruz Biotechnology), ABCG2 (sc-58224, Santa Cruz Biotechnology), horseradish peroxidase (HRP)-conjugated β-actin loading control monoclonal antibody (sc-47778, Santa Cruz Biotechnology), and HRP-conjugated antimouse IgG secondary antibody (ab6728, Abcam). Cells were collected from 6-well plates (5 × 10^4^ cells/well), which were treated with or without different concentrations of compound **2** for 48 h. Later, cells were treated with lysis buffer containing 292 mM saccharose, 100 mM Tris (pH 7.6), 5 mM magnesium chloride (MgCl_2_), 0.1% (w/v) sodium dodecyl sulfate (SDS), 1% (w/v) nonyl phenoxypolyethoxylethanol (NP40), 5 mM ethylenediamine tetraacetic acid (EDTA), and protease inhibitor (P9599, Sigma-Aldrich). The total protein concentrations were quantified with a Pierce BCA protein assay kit (23225, Thermo Scientific). Protein separation was performed using 10% sodium dodecyl sulfate-polyacrilamide gel (SDS-PAGE), and the target protein was transferred from the gel to a PVDF membrane. The transferred membrane was then washed in Tris-buffered saline with Tween 20 (TBS-T) and incubated in BSA 2.5% (Abcam) for 2 h at room temperature. After TBS-T washes, the membrane was subjected to an overnight incubation with primary antibodies at 4 °C. Following an additional TBS-T wash, the membrane was subjected to an incubation with secondary antibodies, subsequently followed by color development with a chromogenic solution of 3-amino-9-ethylcarbazole for a duration of 10 min (A5754–10G, Sigma-Aldrich). Then, the bands were subjected to analysis using ImageJ software.

## Supplementary Material



## Data Availability

Data will be made available under request when solicited accordingly. The NMR data for compound **1** has been deposited (accession number: NP0352024) at NP-MRD (https://np-mrd.org).

## Notes

Supplementary data (PDF) associated to this article can be accessed free of charge in the online version at . The Supporting Information is available free of charge at https://pubs.acs.org/doi/10.1021/acs.jnatprod.5c01273.

Photographs of *Ipomoea funis* flowers, as well as TLC and HPLC chromatogram of fraction F3. Purification process of funisin I (**1**) by recycling HPLC; proposed mechanism for the intramolecular transesterification between funisin I (**1**) and intrapilosin V (**3**); NMR and HRMS spectra for the isolated compounds **1**–**3** and their derivatives, in addition to tables for NMR data for compounds **5**, **7**, and **8**; UV and IR spectra for **1**; cytotoxicity of vinblastine and podophyllotoxin against MCF-7 and MCF-7/Vin after 72 h (PDF)
