# Supplementary material for: Resin Glycosides from Ipomoea funis as Inhibitors of P‑Glycoprotein in Multidrug-Resistant Breast Carcinoma Cells
Source: J Nat Prod. 2026 Jan 8;89(1):220–32. doi: 10.1021/acs.jnatprod.5c01273 (PMC12836347; doi:10.1021/acs.jnatprod.5c01273)
Supplement: Supplementary file 1 [file np5c01273_si_001.pdf]

Supporting Information

Resin Glycosides from *Ipomoea funis* as Inhibitors of P-glycoprotein in Multidrug-Resistant Breast Carcinoma Cells

Pedro de Jesús Flores-Tafoya[a], Jennifer Alexis Rojas-Morales[a,b], Adriana Carolina Hernández-Rojas[a], Mabel Fragoso-Serrano[a], Nohemí Salinas-Jazmín[c], Elihú Bautista[b], Martha Lydia Macías-Rubalcava[d], Rogelio Pereda-Miranda[a,]*

[a]Departamento de Farmacia, Facultad de Química and Programa de Maestría y Doctorado en Ciencias Químicas, Universidad Nacional Autónoma de México, Ciudad Universitaria, Ciudad de México 04510, México.
[b]Secretaría de Ciencia, Humanidades, Tecnología e Innovación and División de Biología Molecular, Instituto Potosino de Investigación Científica y Tecnológica A. C., San Luis Potosí, S.L.P., 78216, México.
[c]Departamento de Farmacología, Facultad de Medicina. Universidad Nacional Autónoma de México, 04510, México.
[d]Departamento de Productos Naturales. Instituto de Química, Universidad Nacional Autónoma de México, Ciudad Universitaria, Ciudad de México, 04510, México.

*Corresponding Author

Email address: pereda@unam.mx

Table of contents

Figure S1. *Ipomoea funis* Cham. & Schltdl., Convolvulaceae and TLC over Si gel of the total extract 1

Figure S2. ^{1}H-NMR (600 MHz) of fraction F3 2

Figure S3. Chromatogram of fraction F3 3

Figure S4. Purification process of compound **1** by recycling HPLC 4

Figure S5. Isotopic distribution of the $[M + H]^+$ adduct for **1** on positive-ion mode HRESIMS 5

Figure S6. Chemical structure of operculinic acid A (**6**) 6

Figure S7. Operculinic acid A (**6**): A)^{13}C (175 MHz) and B) ^{1}H (700 MHz) NMR spectra in pyridine-d_5 7

Figure S8. ^{1}H-NMR spectrum (700 MHz) of funisin I (**1**) in pyridine-d_5 8

Figure S9. ^{13}C-NMR spectrum (175 MHz) of funisin I (**1**) in pyridine-d_5 9

Figure S10. ^{1}H-^{1}H COSY spectrum (700 MHz) of funisin I (**1**) in pyridine-d_5 10

Figure S11. ^{1}H-^{1}H COSY: expansion for the oligosaccharide core region of funisin I (**1**) in pyridine-d_5 11

Figure S12. TOCSY: expansion for the oligosaccharide core region of funisin I (**1**) in pyridine-d_5 12

Figure S13. ^{1}H-Detected heteronuclear ($^{1}J_{CH}$) correlation (HSQC) spectrum for funisin I (**1**) 13

Figure S14. ^{1}H-Detected heteronuclear ($^{1}J_{CH}$) correlation (HSQC) spectrum for the anomeric signals of funisin I (**1**) 14

Figure S15. ^{1}H-Detected heteronuclear ($^{2,3}J_{CH}$) correlation (HMBC) spectrum of funisin I (**1**) 15

Figure S16. HMBC expansion: connectivity assignments for the glycosylation sequence ($^{3}J_{CH}$) of funisin I (**1**) 16

Figure S17. HMBC expansion: connectivity assignments for the sites of esterification ($^{2,3}J_{CH}$) of funisin I (**1**) 17

Figure S18. Purginoside I (**4**): A) ^{1}H (500 MHz) and B) ^{13}C (125 MHz) NMR spectra in pyridine-d_5 18

Figure S19. ^{1}H-NMR spectrum (500 MHz) of acutacoside F (**5**) in pyridine-d_5 19

Figure S20. ^{1}H-detected heteronuclear correlation (HMBC) spectrum for acutacoside F (**5**) 20

Figure S21. ^{1}H NMR pattern for the multiplicity and chemical shifts of the CH_2 in α position to the aglycone carbonyl C-1 21

Figure S22. Chemical structures for peracetylated derivatives **7** and **8** 22

Figure S23. ^{1}H-NMR spectrum (700 MHz) in pyridine-d_5 of the peracetylated mixture of compounds **7** and **8** 23

Figure S24. ^{1}H-Detected heteronuclear ($^{2,3}J_{CH}$) correlation (HMBC) spectrum in pyridine-d_5 of the peracetylated mixture of compounds **7** and **8** 24

Figure S25. HMBC (**$^{3}J_{CH}$**) connectivity expansion for the esterification sequence in the peracetylated mixture for acetylated derivatives 25

Figure S26. Proposed mechanism for the intramolecular transesterification between funisin I (**1**) and intrapilosin V (**3**) 26

Figure S27. Cytotoxicity of vinblastine and podophyllotoxin against MCF-7 and MCF-7/Vin after 72 h 27

Figure S28. Images of vinblastine-sensitive MCF-7 cells and vinblastine-resistant MCF-7/Vin cells after 72 hours of treatment with vinblastine (Vin) 28

Figure S29. Activation of caspase-3 protein in MCF-7/Vin cells 29

Figure S30. UV spectrum for funisin I (**1**) 30

Figure S31. FTIR spectrum for funisin I (**1**) 31

Figure S32. 1D-NMR spectra in pyridine-d_5 for intrapilosin I (**2**). ^{1}H (A, 700 MHz) and ^{13}C (B, 175 MHz) 32

Figure S33. 1D-NMR spectra in pyridine-d_5 for intrapilosin V (**3**). ^{1}H (A, 700 MHz) and ^{13}C (B, 175 MHz) 33

Table S1. NMR Spectroscopic Data (700 MHz, pyridine-d_5) of compound **7** 34

Table S2. NMR Spectroscopic Data (700 MHz, pyridine-d_5) of compound **8** 35

Table S3. Cytotoxicity for isolated pure compounds (**1**-**3**) and control drugs in standard SRB assays 36

Table S4. NMR Spectroscopy Data (700 MHz, pyridine-d_5) of operculinic acid A (**6**) 37

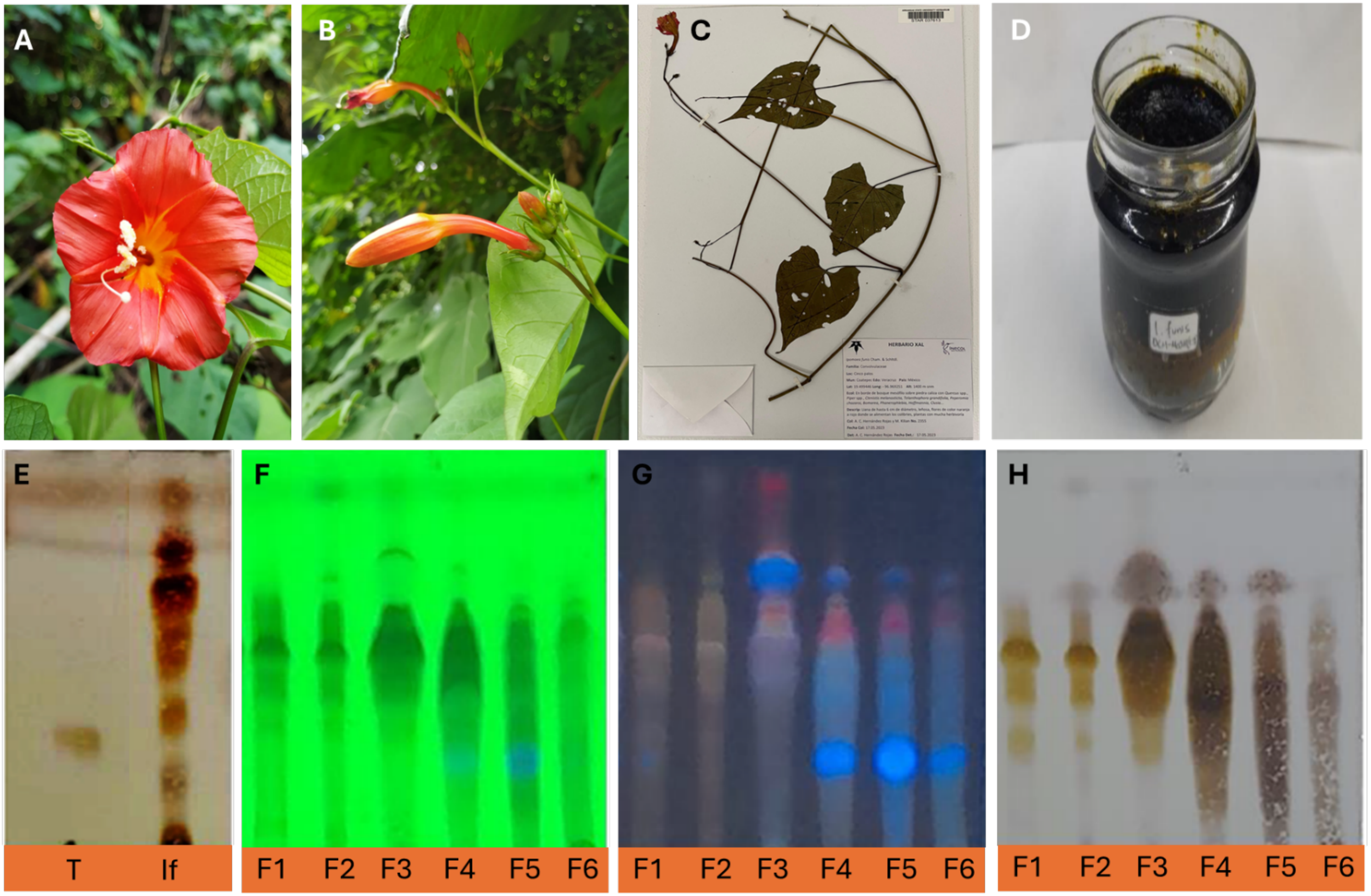

Figure S1. *Ipomoea funis* Cham. & Schltdl., Convolvulaceae: A) flower; B) side profile of the distinct awn on the abaxial sepal surface of Quamoclit spp; C) officially recognized specimen by the XAL (Mexico) and STAR (USA) herbariums. D) CH_2Cl_2-MeOH dried extract. E) TLC over Si gel of the total extract from *I. funis* (If) against tricolorin A (T), a resin glycoside from *Ipomoea tricolor*; CH_2Cl_2-MeOH (9:1). F) TLC of the resulting CC six fractions (F1-F6) under UV light at 254 nm. G) TLC under UV light at 365 nm. G) TLC revealed with acidified cerium sulfate and heating.

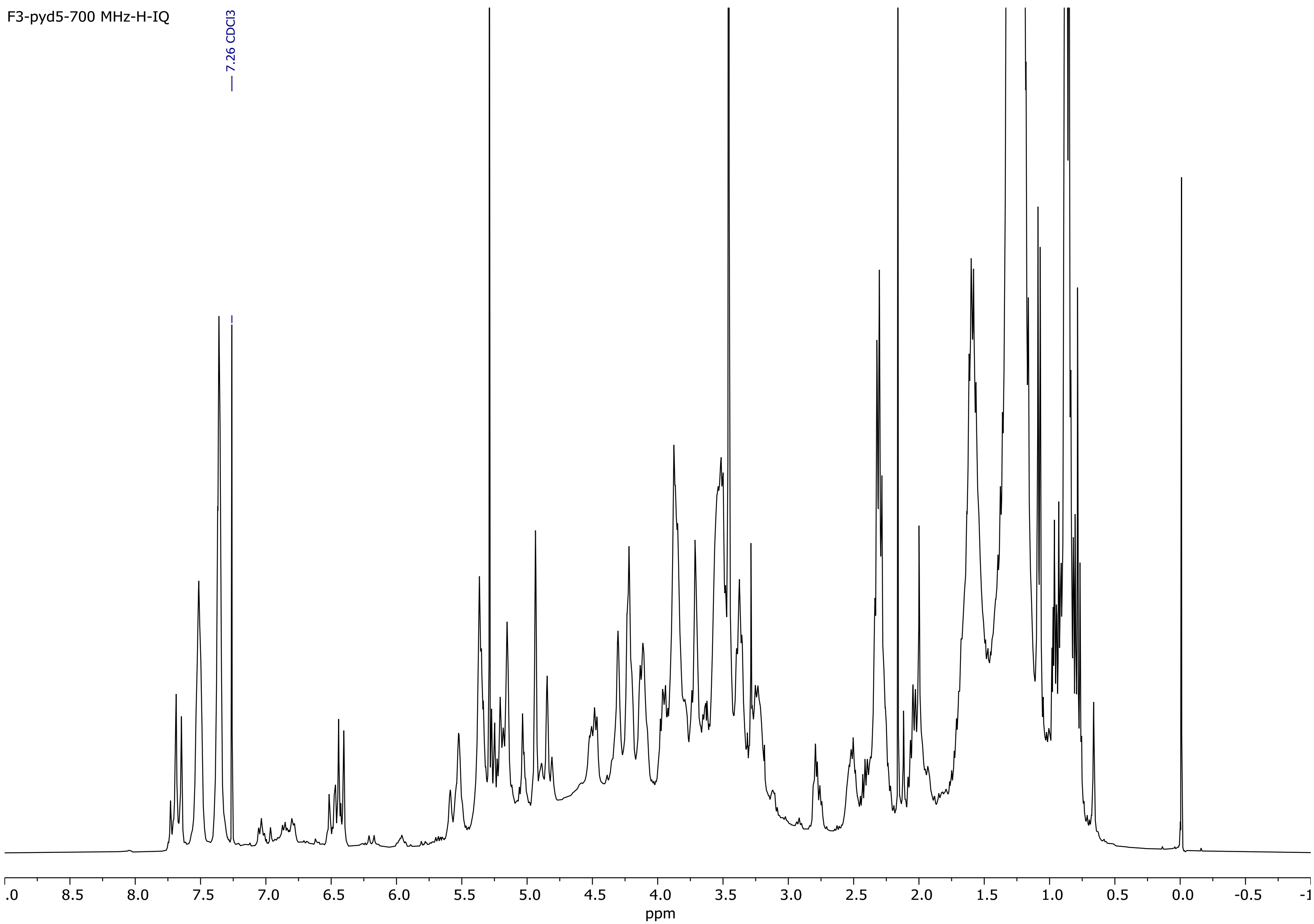

Figure S2. ^{1}H- NMR (600 MHz) of fraction F3 in chloroform-*d*. The spectra was recorded in a JEOL ECZ600R spectrometer on microtubes (2.5 mm × 50 mm; 200 μL).

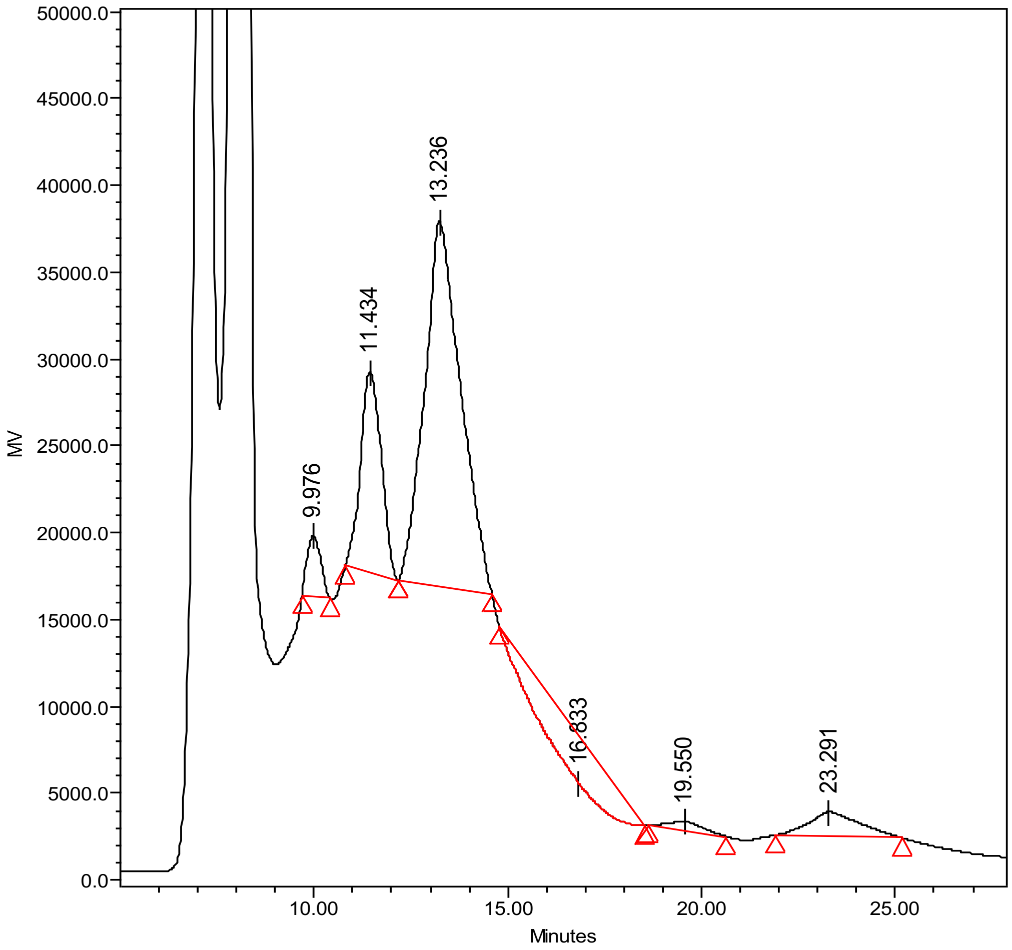

Figure S3. Chromatogram of fraction F3 displaying the isolated peaks and their retention times (t_R) by reversed-phase HPLC under refractive-index detection. Conditions of the analysis were as follows: NH_2 column (19 × 150 mm, 7 µm, Waters *µ*Bondapak); flow rate: 5 mL/min; mobile phase: CH_3CN-MeOH (9:1); sample injection volume: 500 µL (50 mg in THF).

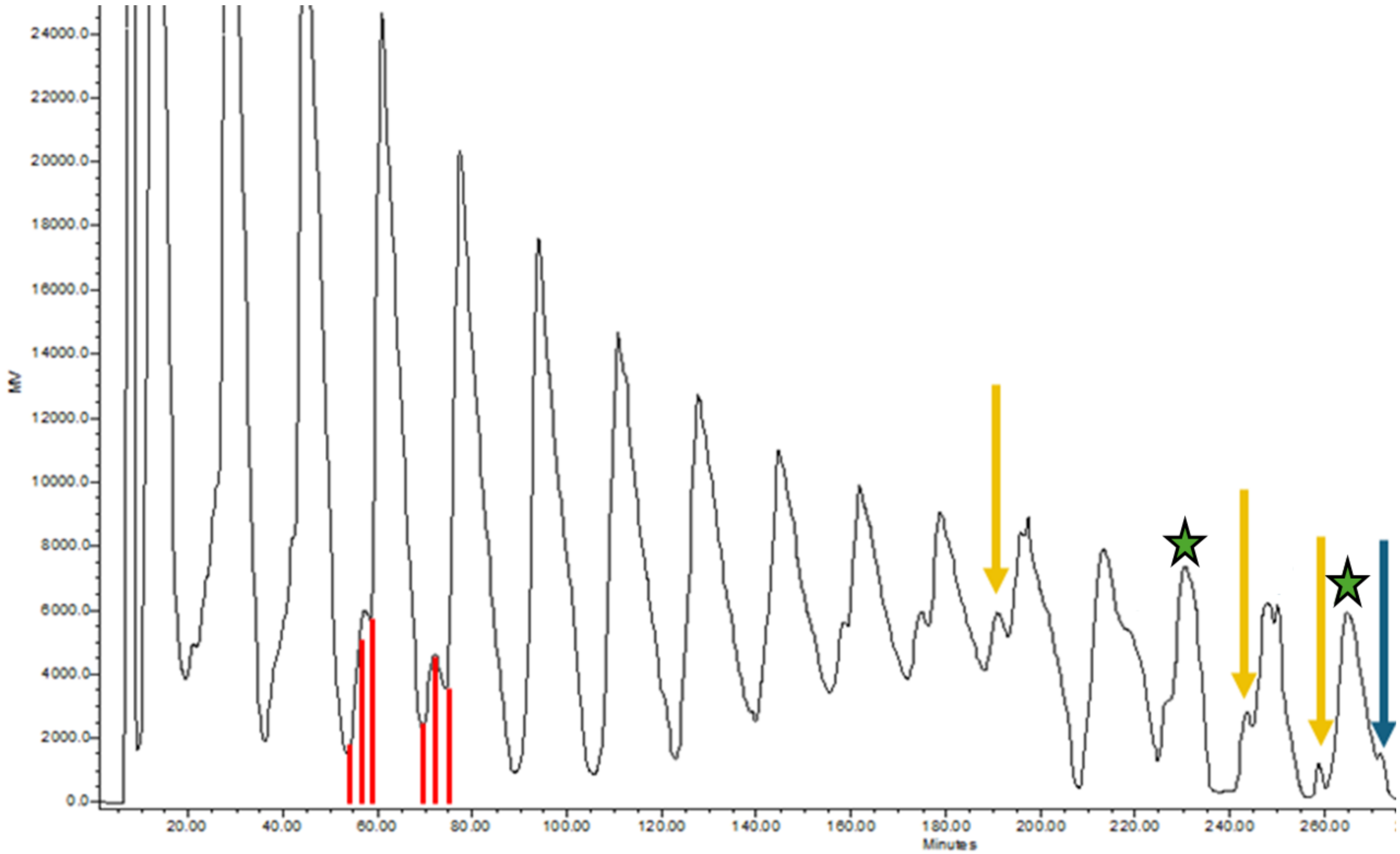

Figure S4. Purification process of compound **1** by recycling HPLC. Red stripes represent initial impurities while starred peaks denote the final recycled and collected peak characterized as compound **1**. Yellow arrows indicate complementary shaved peaks for minor resin glycosides that were discarded during the isolation procedure. Blue arrows afforded supplementary amounts.

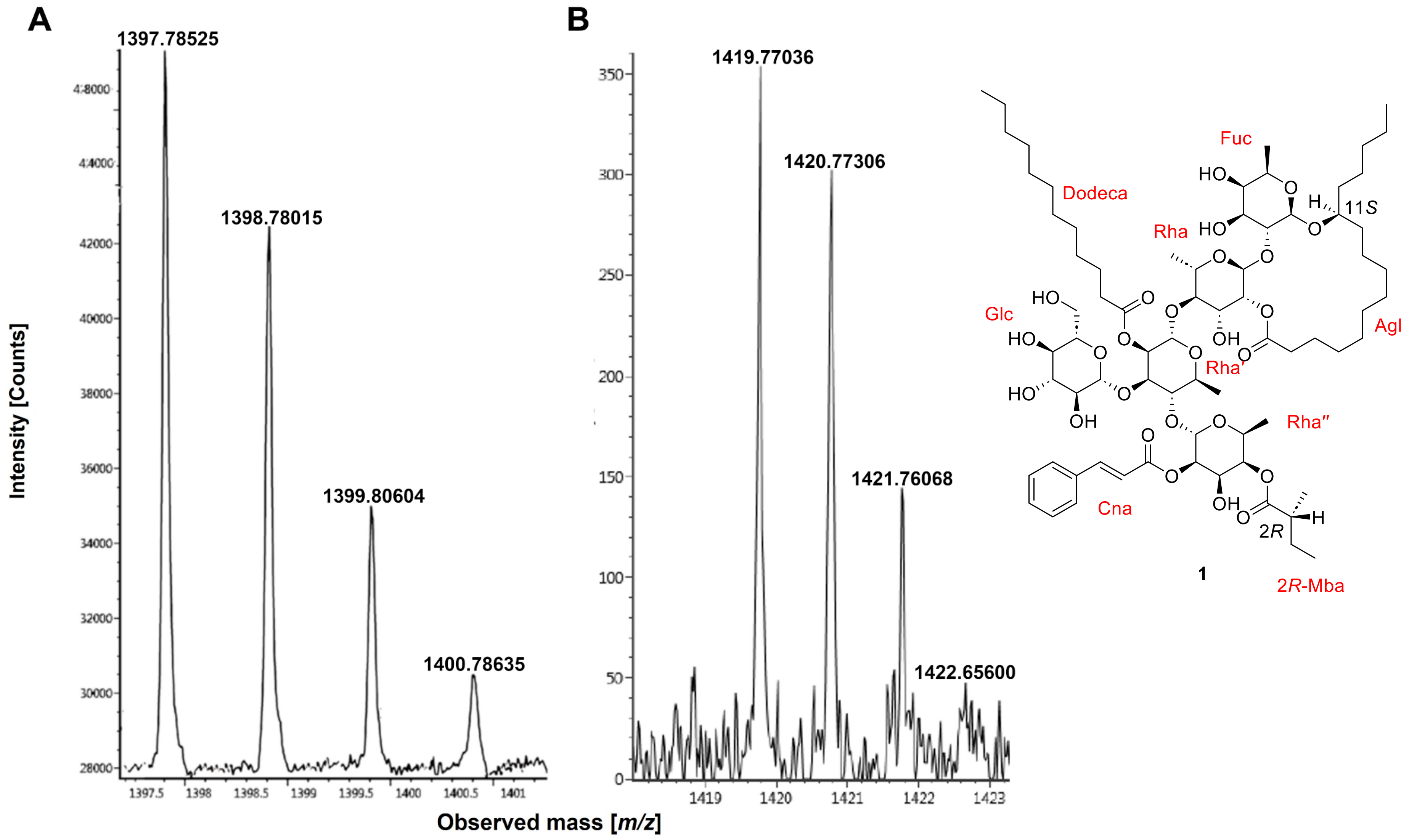

Figure S5. Isotopic distribution of the [M + H]$^+$ (A: 150 ng/mL MeCN/MeOH, formic acid 0.1%) and [M + Na]$^+$ (B: 1 µg/mL MeCN/MeOH) adduct cations for funisin I (**1**) on positive-ion mode HRESIMS.

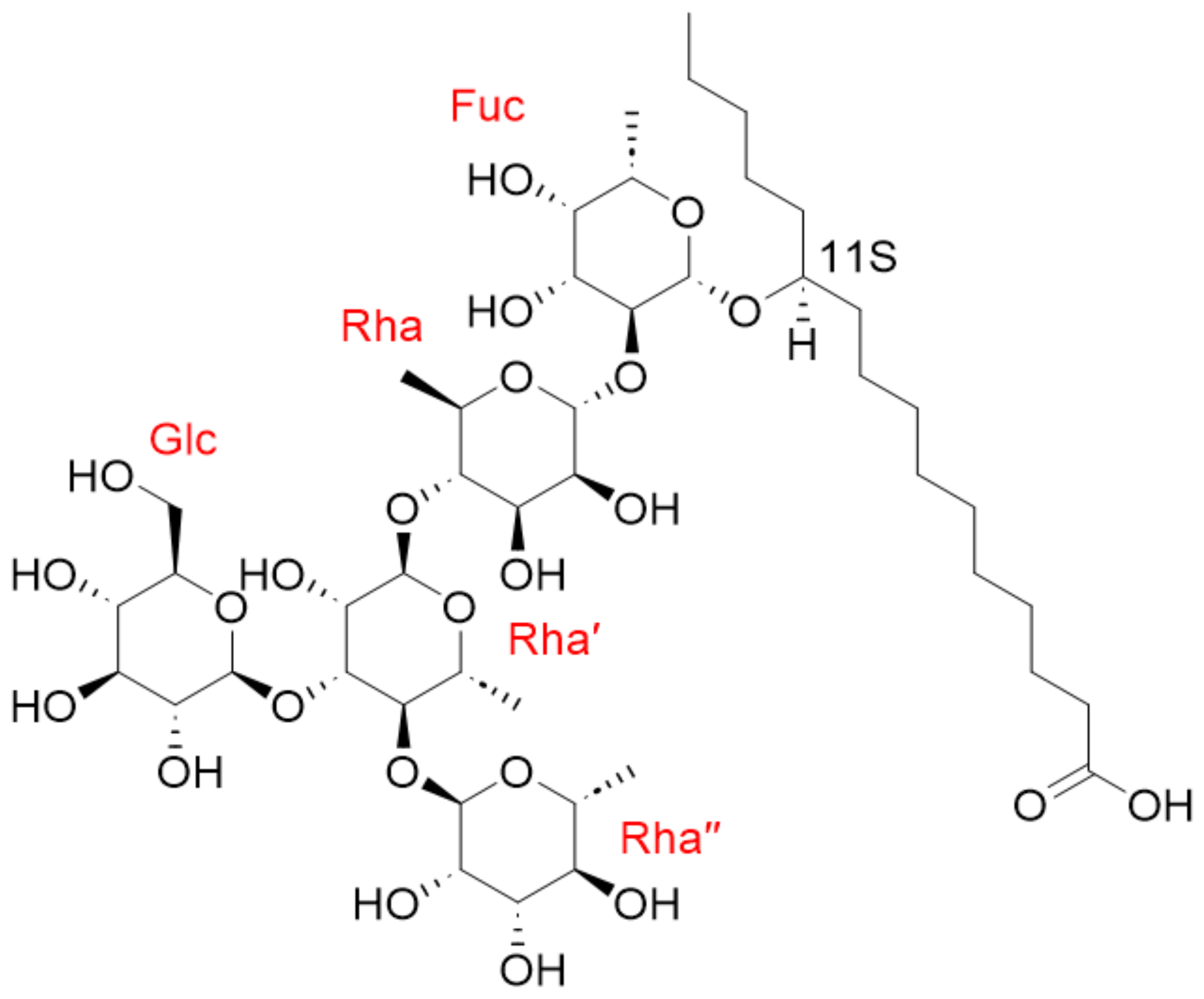

Figure S6. Chemical structure of operculinic acid A (**6**).

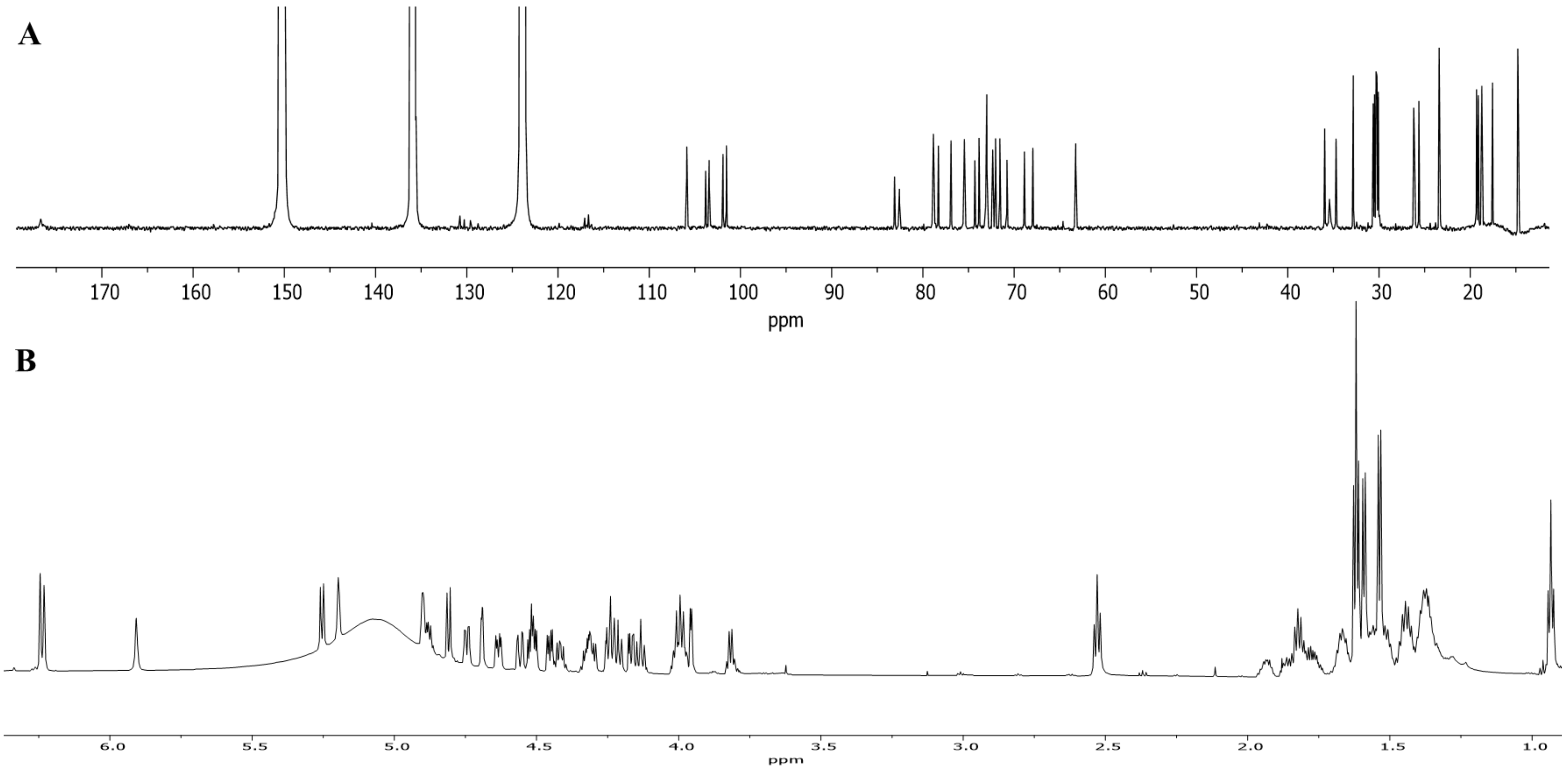

Figure S7. Operculinic acid A (**6**): A)^{13}C (175 MHz) and B) ^{1}H (700 MHz) NMR spectra in pyridine-d_5.

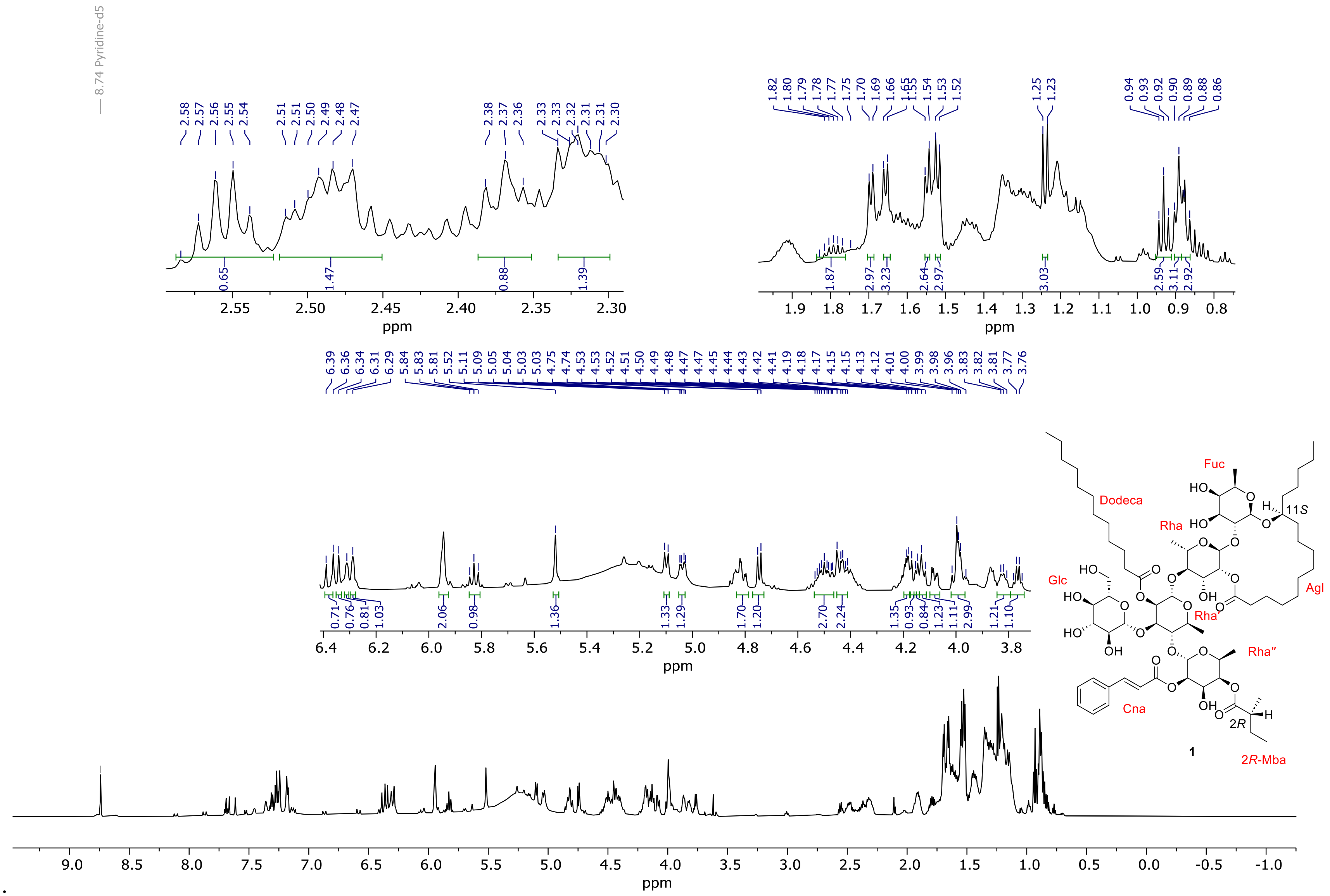

Figure S8. ^{1}H-NMR spectrum (700 MHz) of funisin I (**1**) in pyridine-d_5.

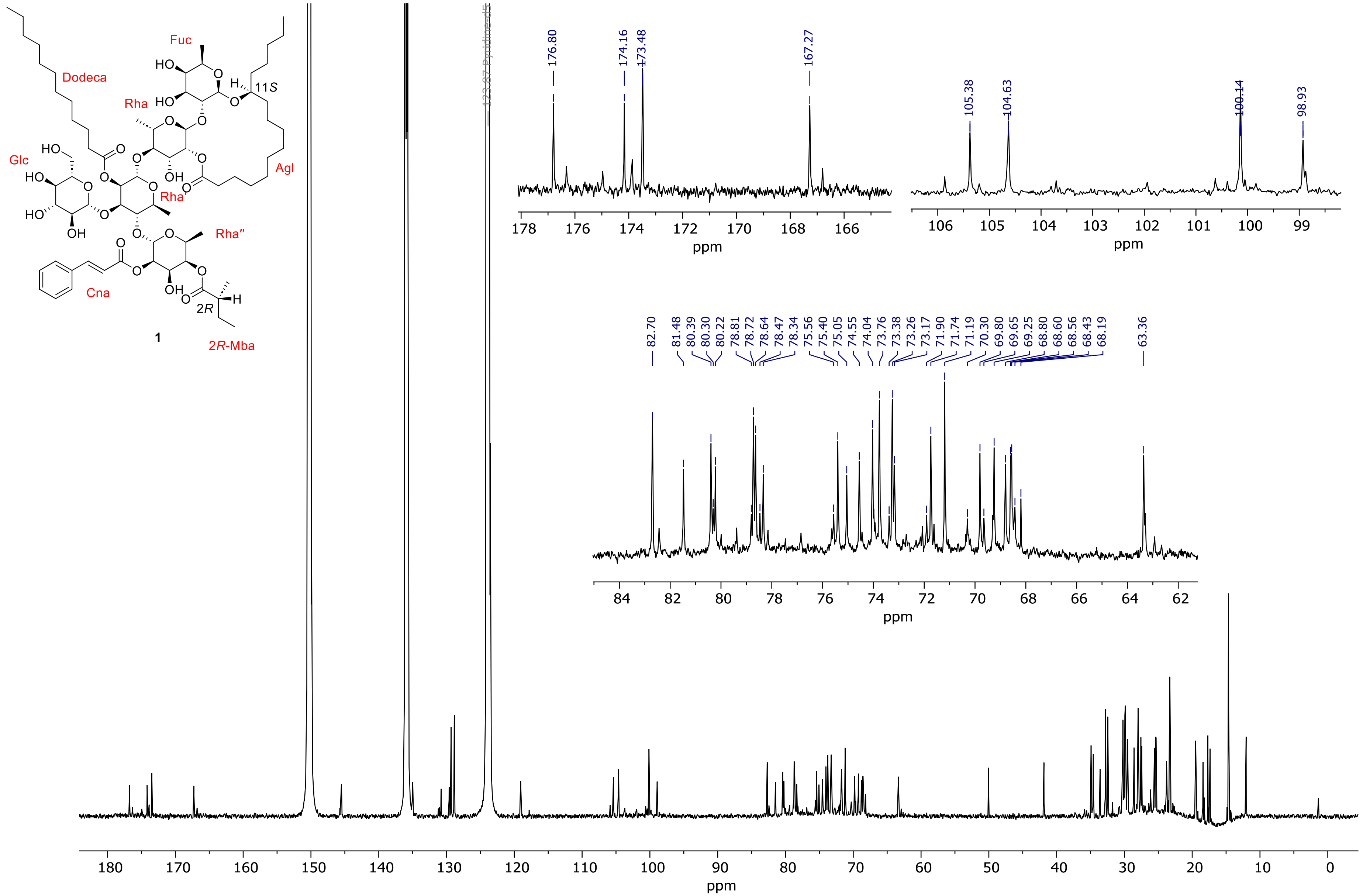

Figure S9. ^{13}C-NMR spectrum (175 MHz) of funisin I (**1**) in pyridine-d_5.

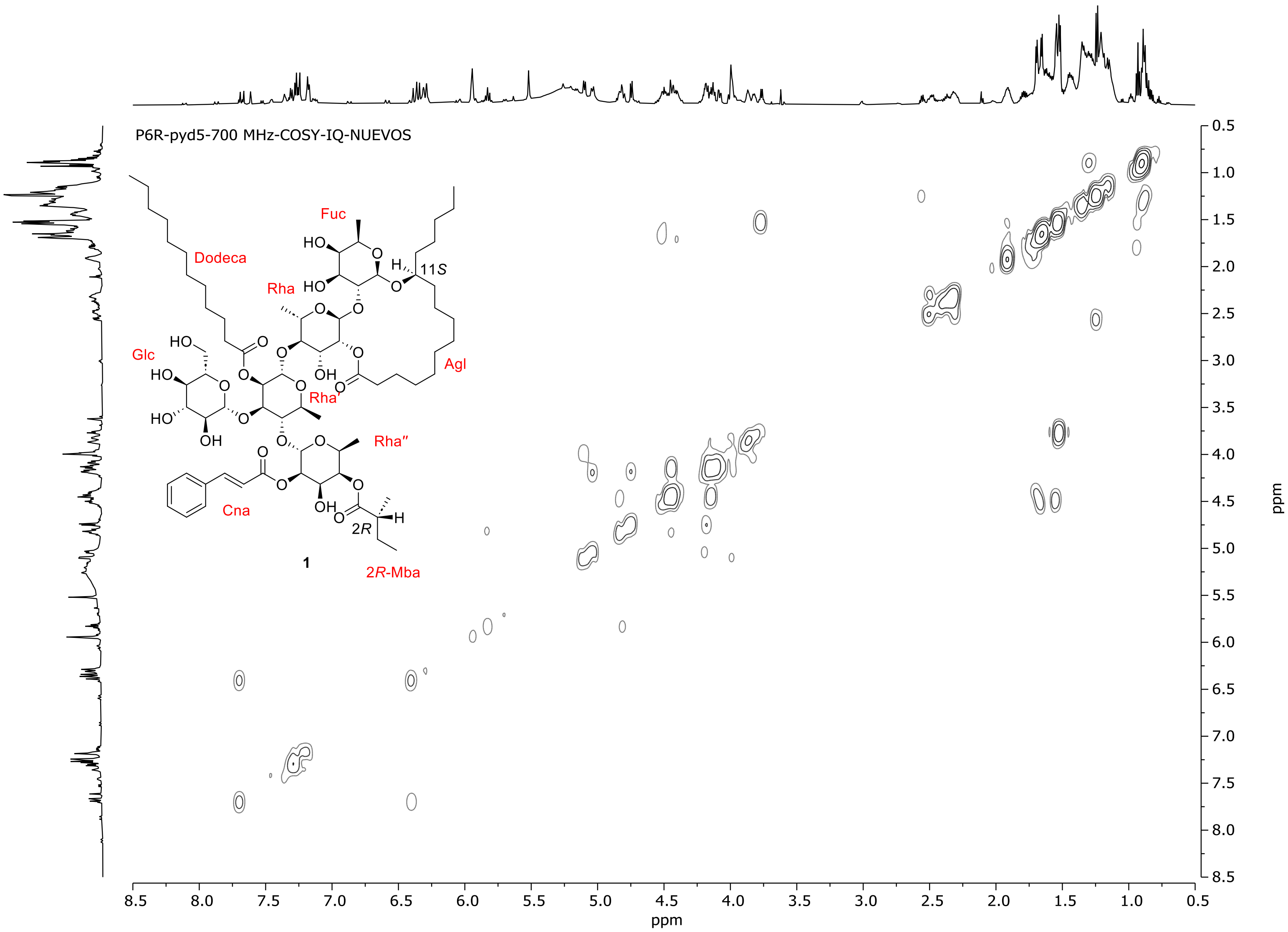

Figure S10. ^{1}H-^{1}H COSY spectrum (700 MHz) of funisin I (**1**) in pyridine-d_5.

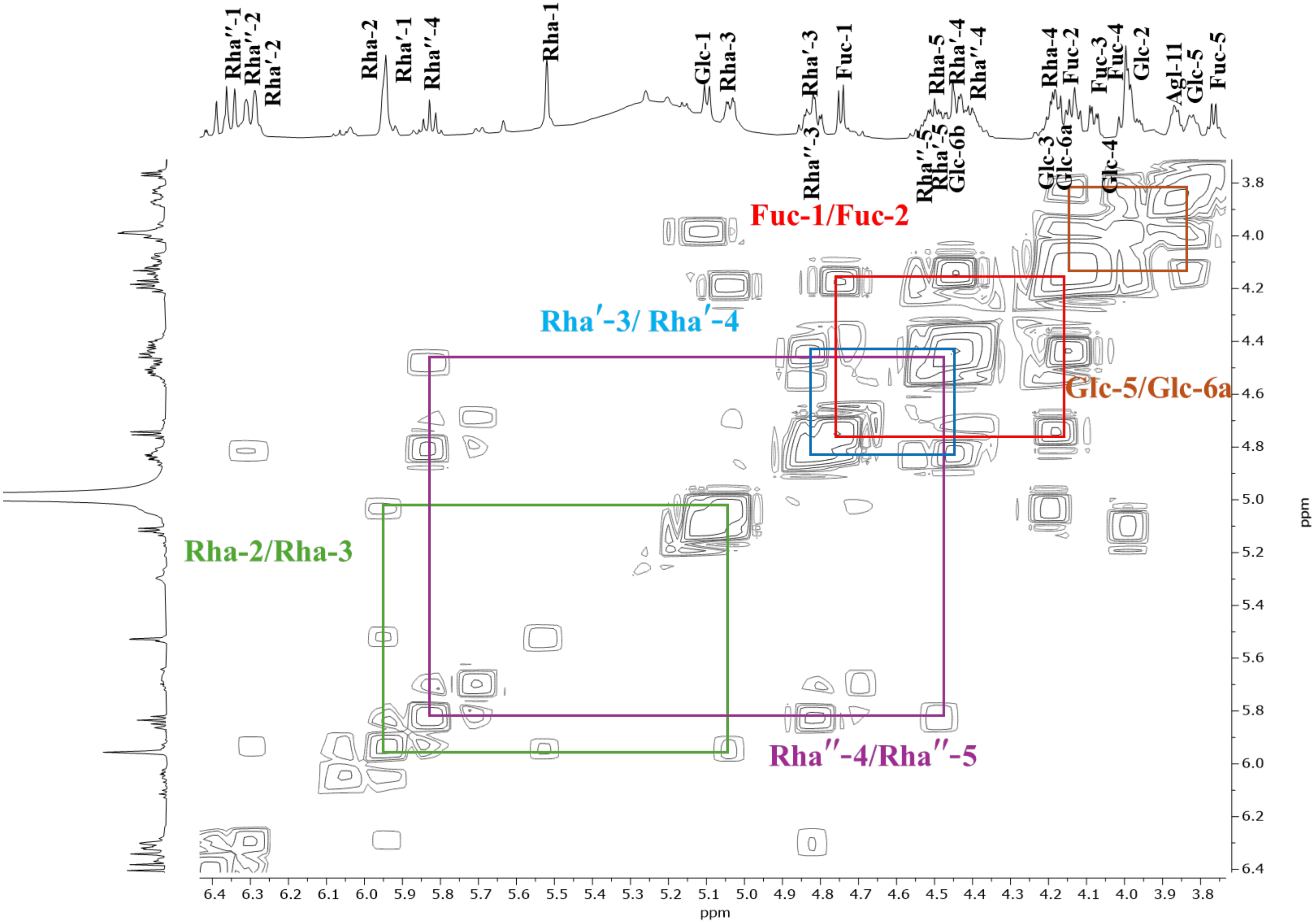

Figure S11. ${}^{1}H$-${}^{1}H$ COSY expansion for the oligosaccharide core region of funisin I (**1**) in pyridine-d_5.

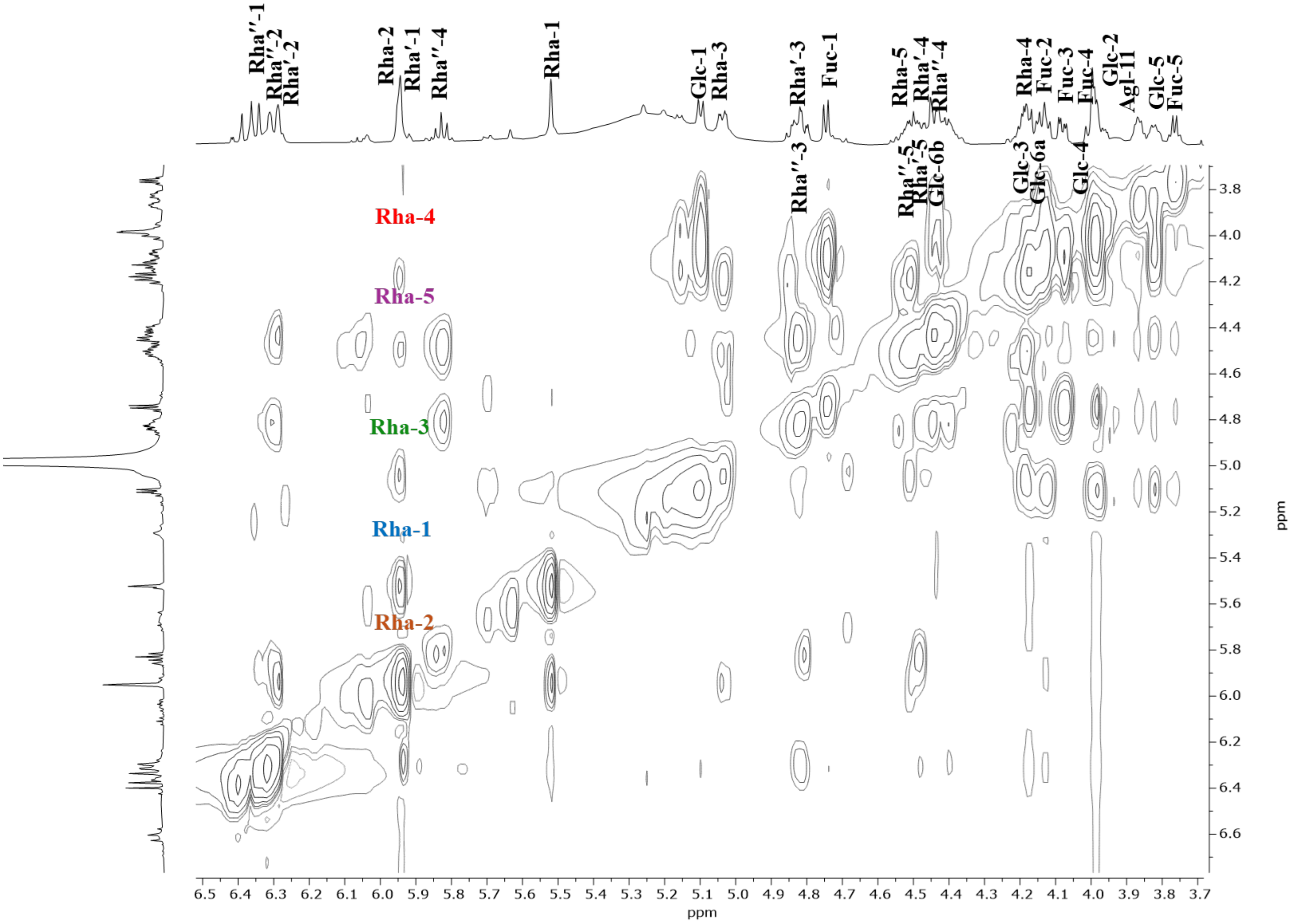

Figure S12. TOCSY expansion for the oligosaccharide core region of funisin I (**1**) in pyridine-d_5.

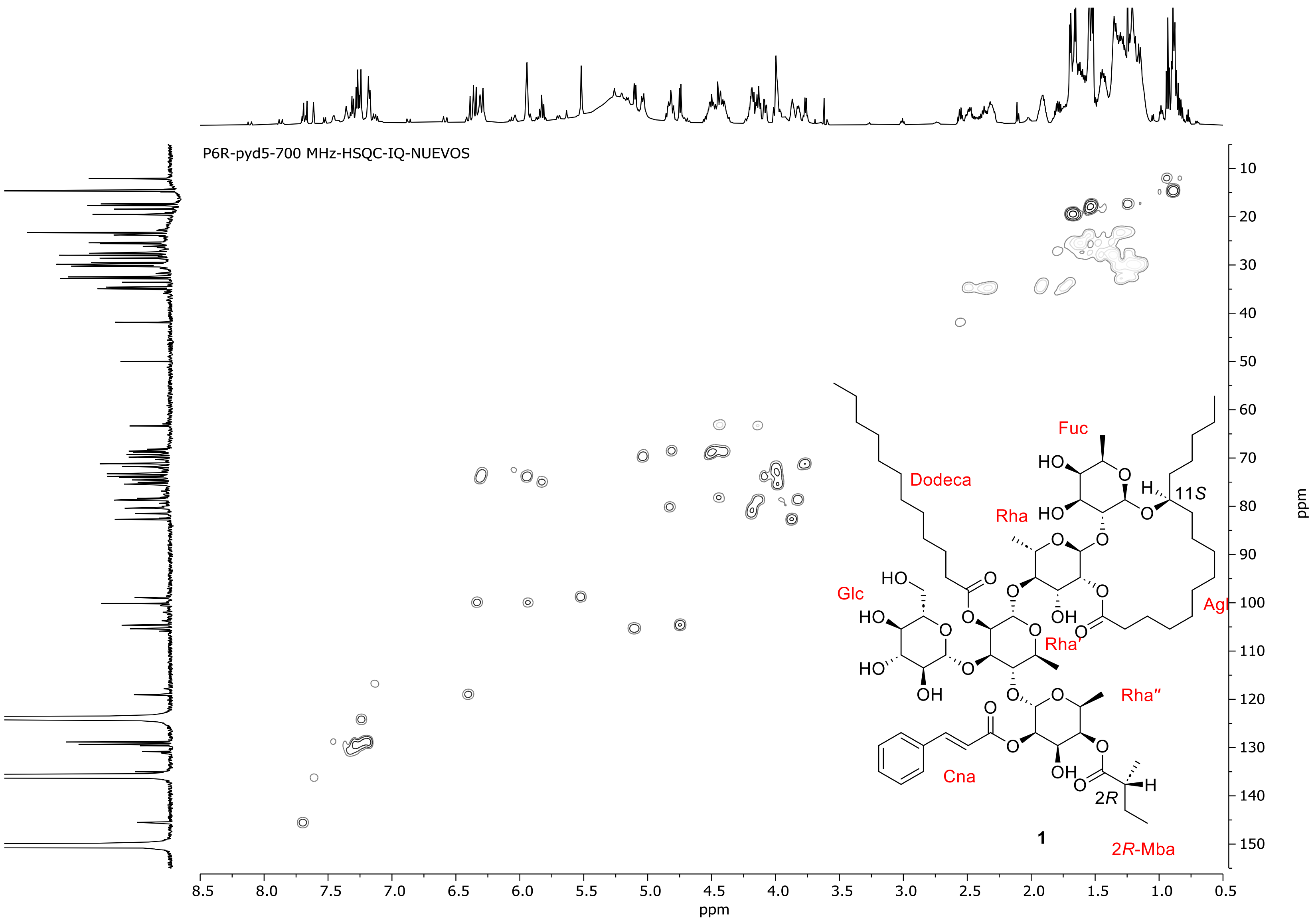

Figure S13. ^{1}H-Detected heteronuclear ($^{1}J_{CH}$) correlation (HSQC) spectrum for funisin I (**1**) with high resolution 1D ^{1}H (700 MHz) and ^{13}C (175 MHz) projections in pyridine-d_5.

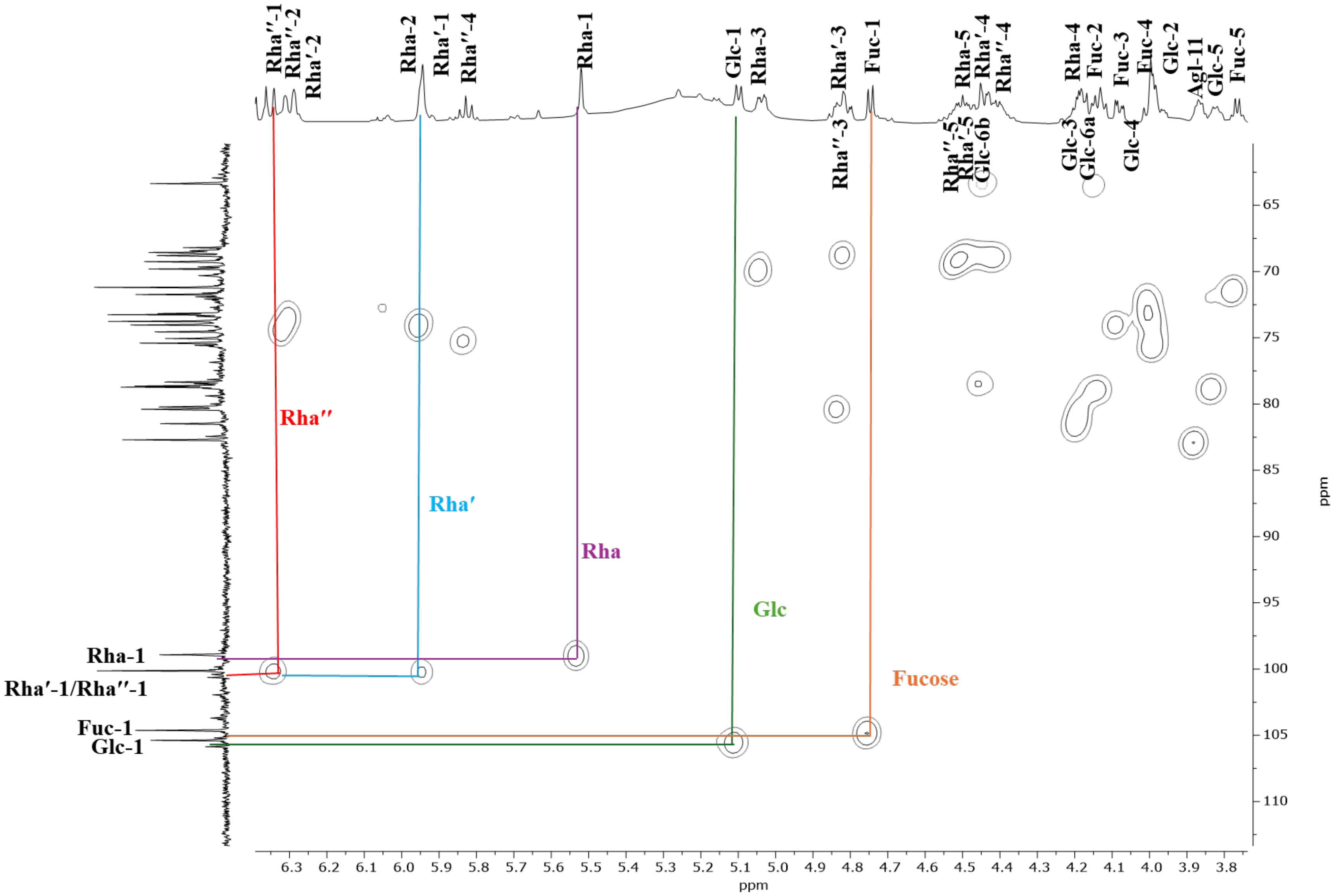

Figure S14. ^{1}H-Detected heteronuclear ($^1J_{CH}$) correlation (HSQC) spectrum for the anomeric signals of funisin I (**1**).

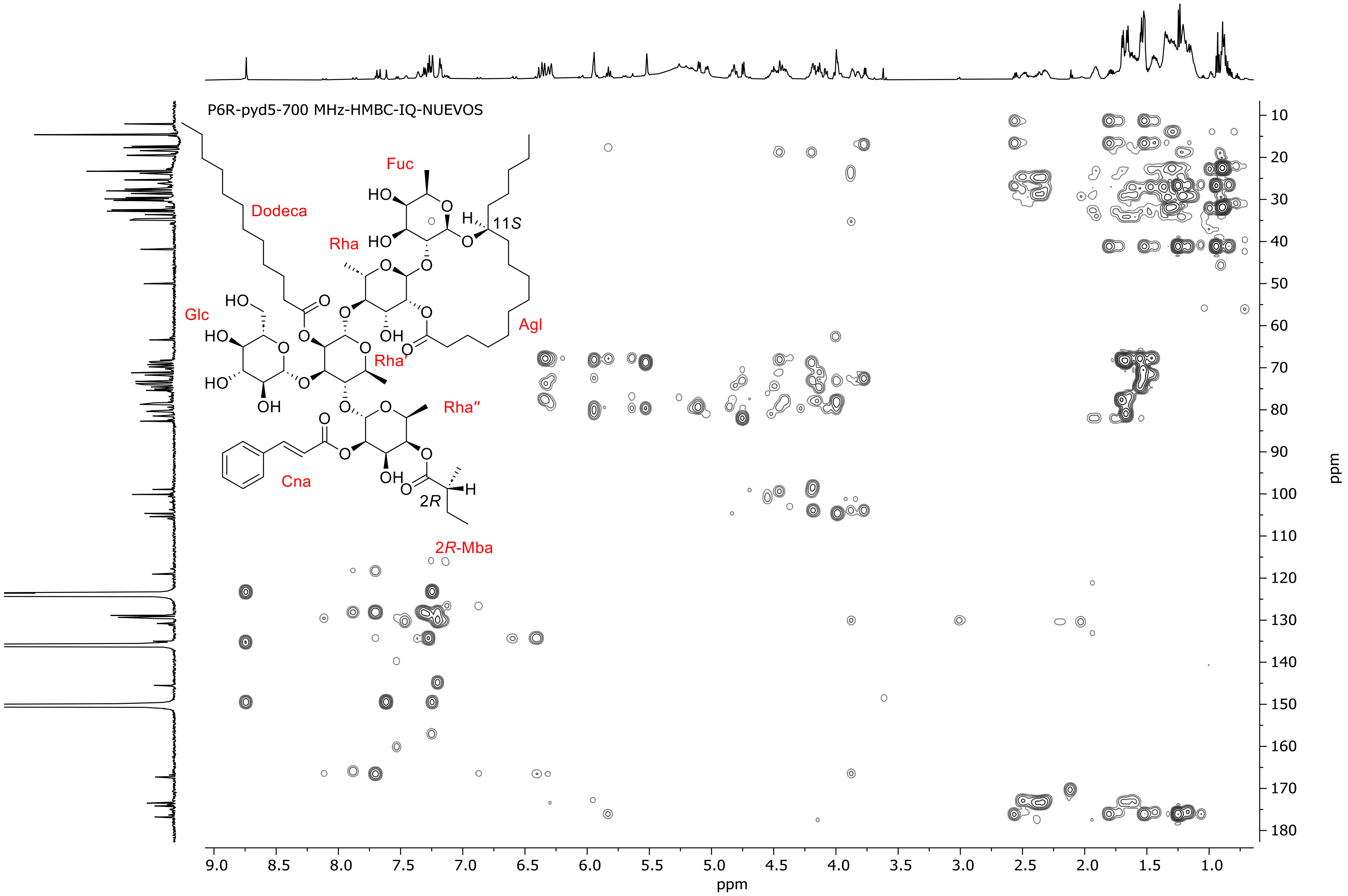

Figure S15. ^{1}H-Detected heteronuclear ($^{2,3}J_{CH}$) correlation (HMBC) spectrum of funisin I **(1)** with high resolution 1D ^{1}H (700 MHz) and ^{13}C (175 MHz) projections in pyridine-d_5.

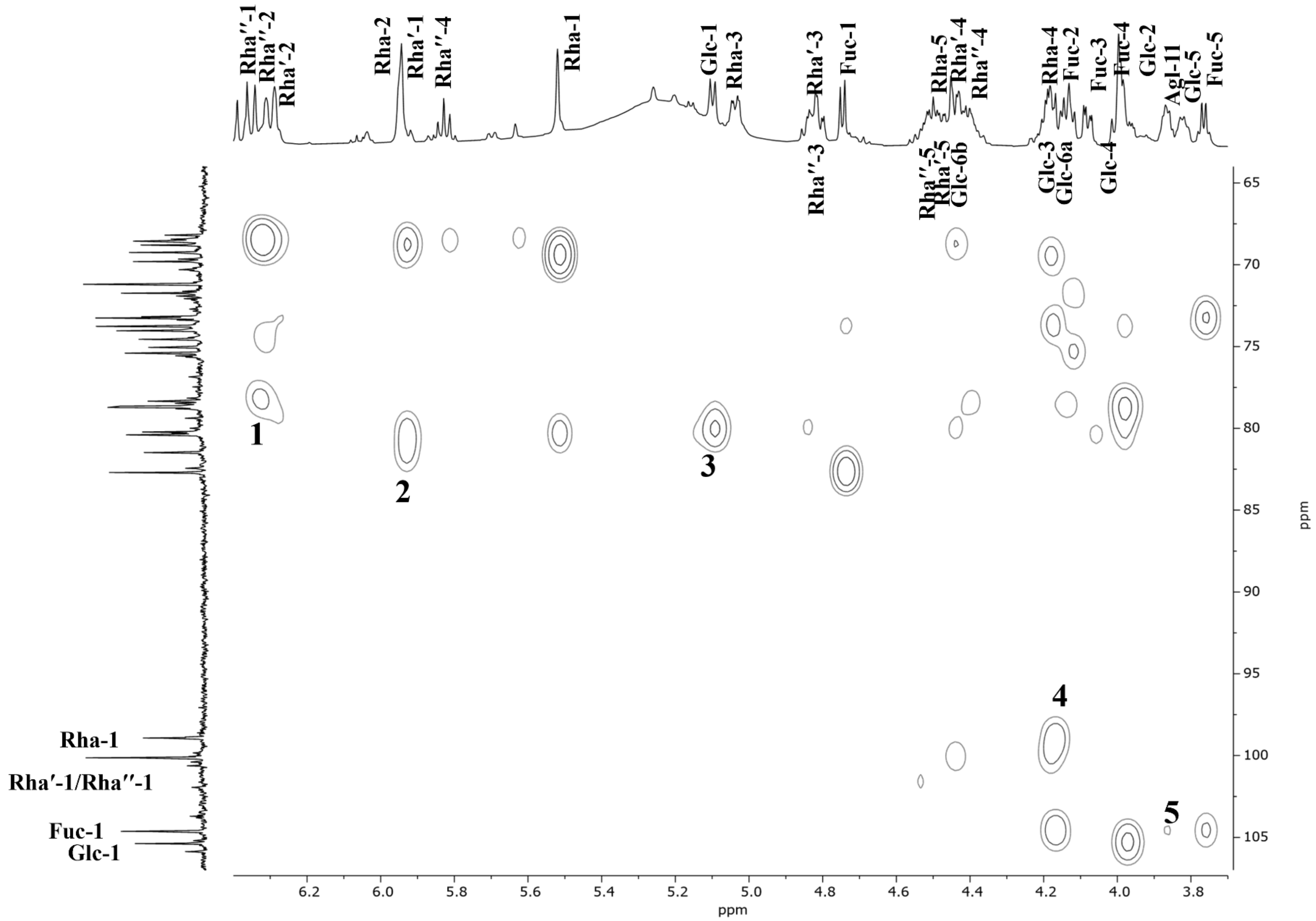

Figure S16. HMBC expansion: connectivity assignments for the glycosylation sequence ($^{3}J_{CH}$) of funisin I (**1**): **1**) Rha′ C-4/Rha″ H-1; **2**) Rha C-4/Rha′ H-1, **3**) Rha′ C-3/Glc H-1, **4**) Rha C-1/Fuc H-2, **5**) Fuc C-1/Agl H-11.

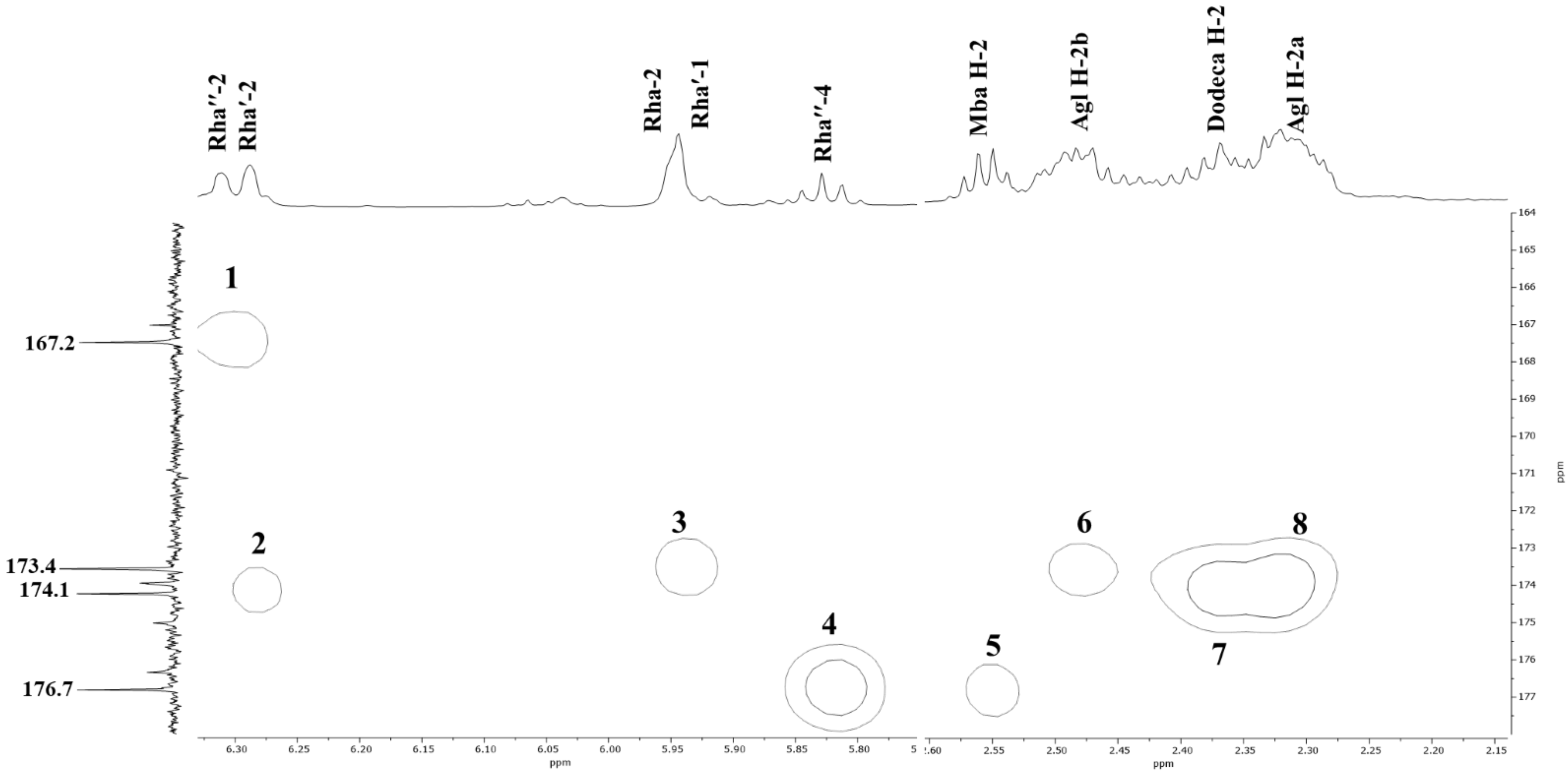

Figure S17. HMBC expansion: connectivity assignments for the sites of esterification ($^{2,3}J_{CH}$) of funisin I (**1**): **1**) Cna C-1/Rha″ H-2, **2**) Dodeca C-1/Rha′ H-2; **3**) Agl C-1/Rha H-2, **4**) Mba C-1/Rha″ H-4; **5**) Mba C-1/Mba H-2, **6**) Agl C-1/Agl H-2b, **7**) Dodeca C-1/ Dodeca H-2, **8**) Agl C-1/ Agl H-2a.

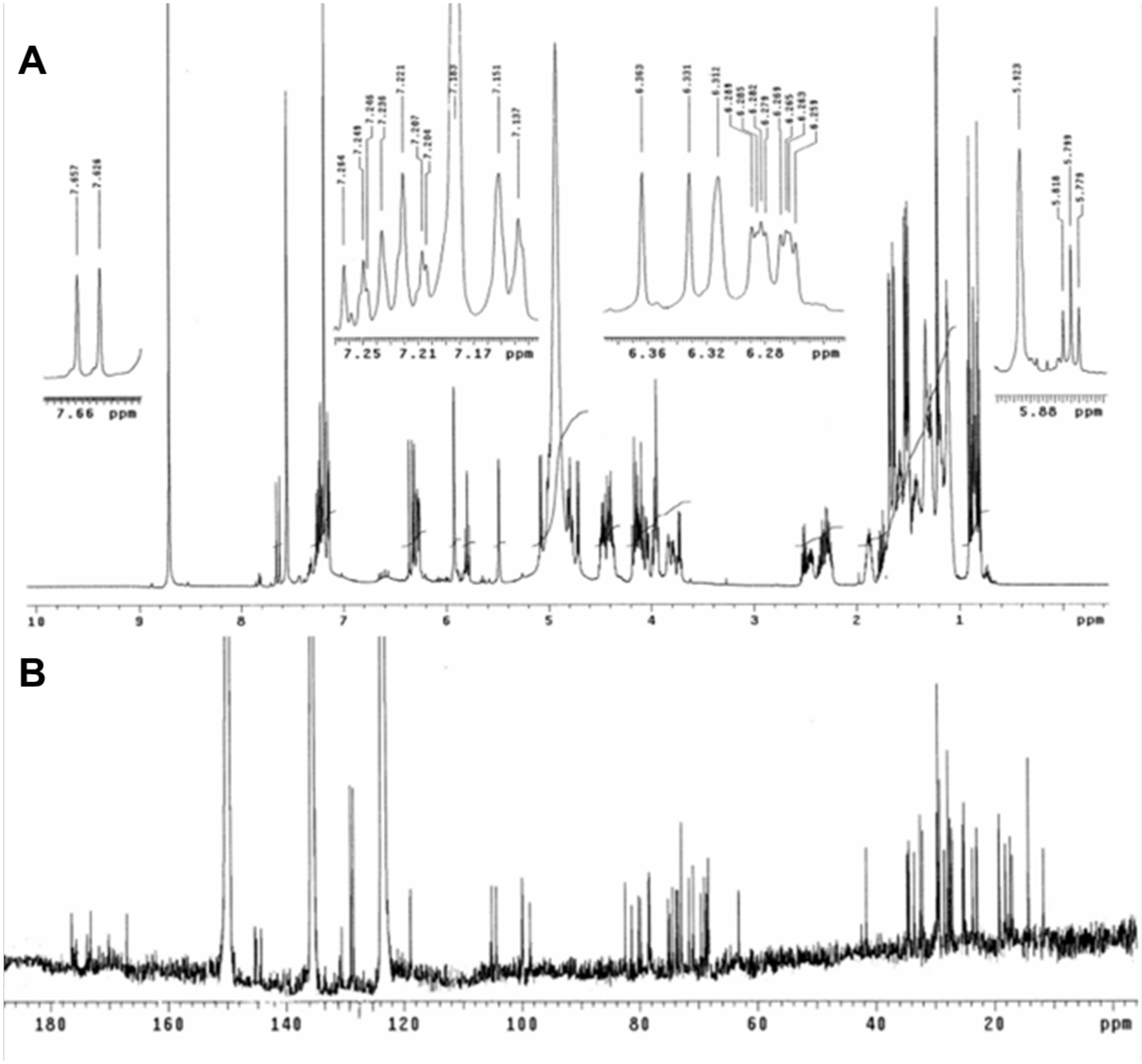

Figure S18. Purginoside I (**4**): A) ^{1}H (500 MHz) and B) ^{13}C (125 MHz) NMR spectra in pyridine-d_5. See, *J. Nat. Prod.* **2011**, 74, 1148–1153; (https://doi.org/10.1021/np200080k).

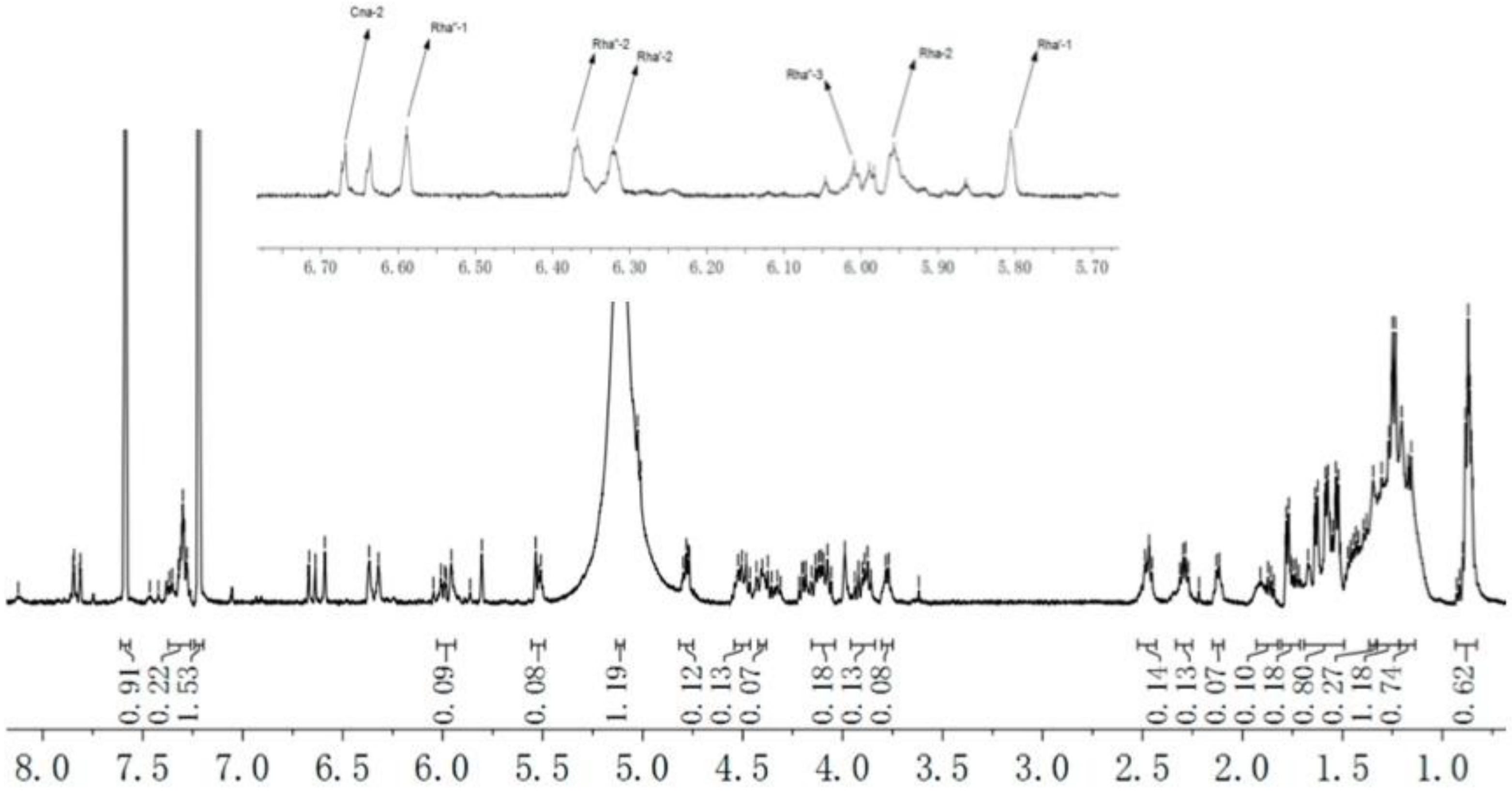

Figure S19. ^{1}H-NMR spectrum (500 MHz) of acutacoside F (**5**) in pyridine-d_5. See, *Molecules* **2017**, *22*(3), 440; https://doi.org/10.3390/molecules22030440.

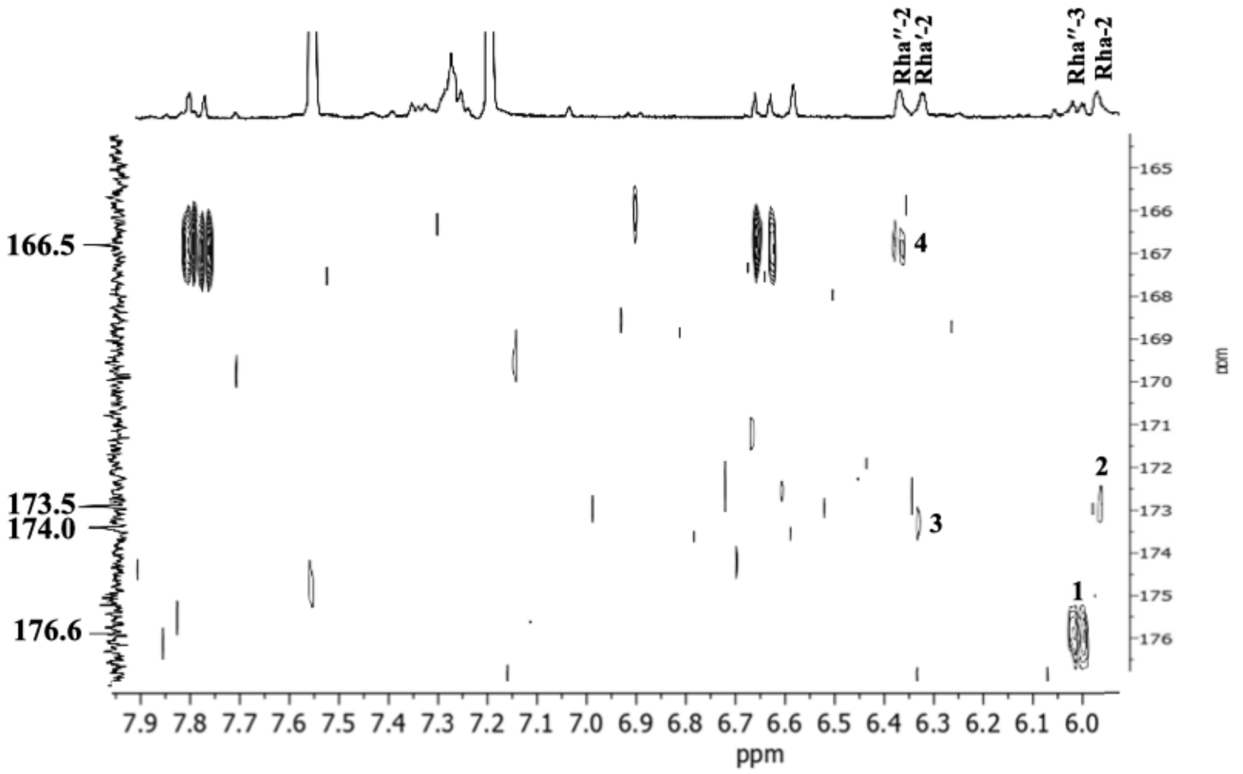

Figure S20. ^{1}H-detected heteronuclear correlation (HMBC) spectrum for acutacoside F (**5**) with high-resolution 1D ^{1}H (500 MHz) and ^{13}C (125 MHz) projections in pyridine-*d*5. Connectivity assignments for the esterification sequence ($^{3}J_{CH}$): **1**, mba C-1/Rha″-3; **2**, Agl C-1/Rha-2; **3**, Dodeca C-1/ Rha′-2; **4**, cna C-1/Rha″-2. Note: the horizontal axis was realigned to match the correct signal assignment, See, *Molecules* **2017**, *22*(3), 440 (https://doi.org/10.3390/molecules22030440).

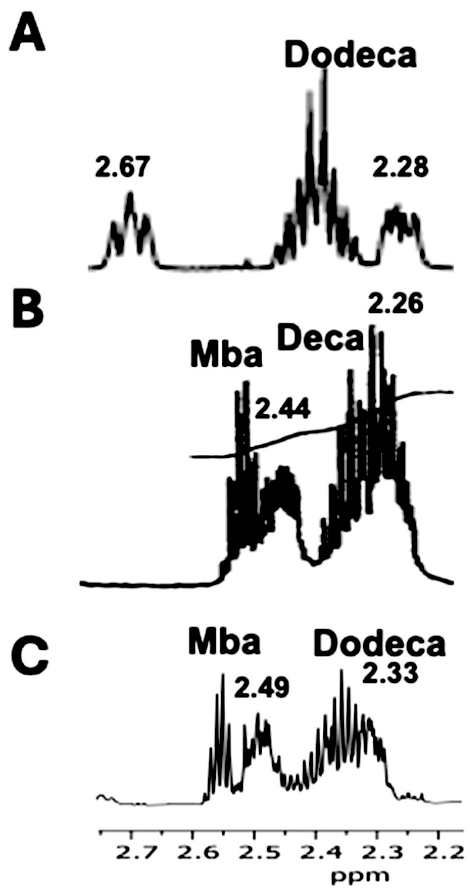

Figure S21. ^{1}H NMR pattern for the multiplicity and chemical shifts of the CH_2 in *α* position to the aglycone carbonyl C-1. The macrolactone ring is closed at Rha C-3 like in batatinoside IX (A). The ring is closed at C-2 in purginoside I (B) and funisin I (C). See, (A) *J. Agric. Food Chem.* **2013**, 61, 39, 9488–9494 (https://doi.org/10.1021/jf402952d); (B) *J. Nat. Prod.* **2011**, 74, 1148–1153 (https://doi.org/10.1021/np200080k).

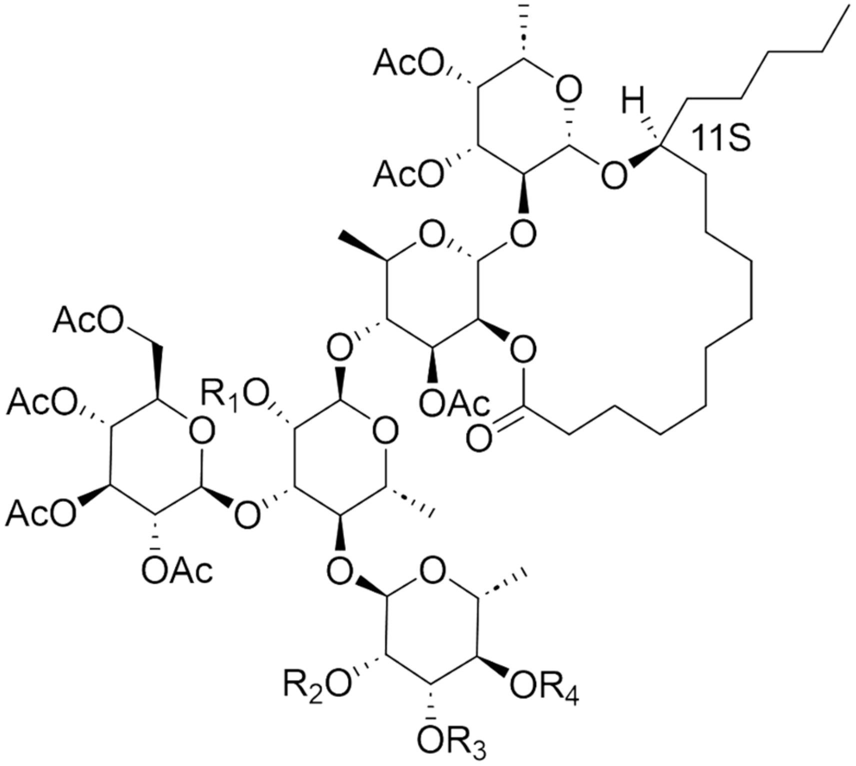

	R1	R2	R3	R4
**7**	Dodeca	Cna	Ac	Mba
**8**	Dodeca	Ac	Cna	Mba

Figure S22. Chemical structures for peracetylated derivatives **7** and **8**.

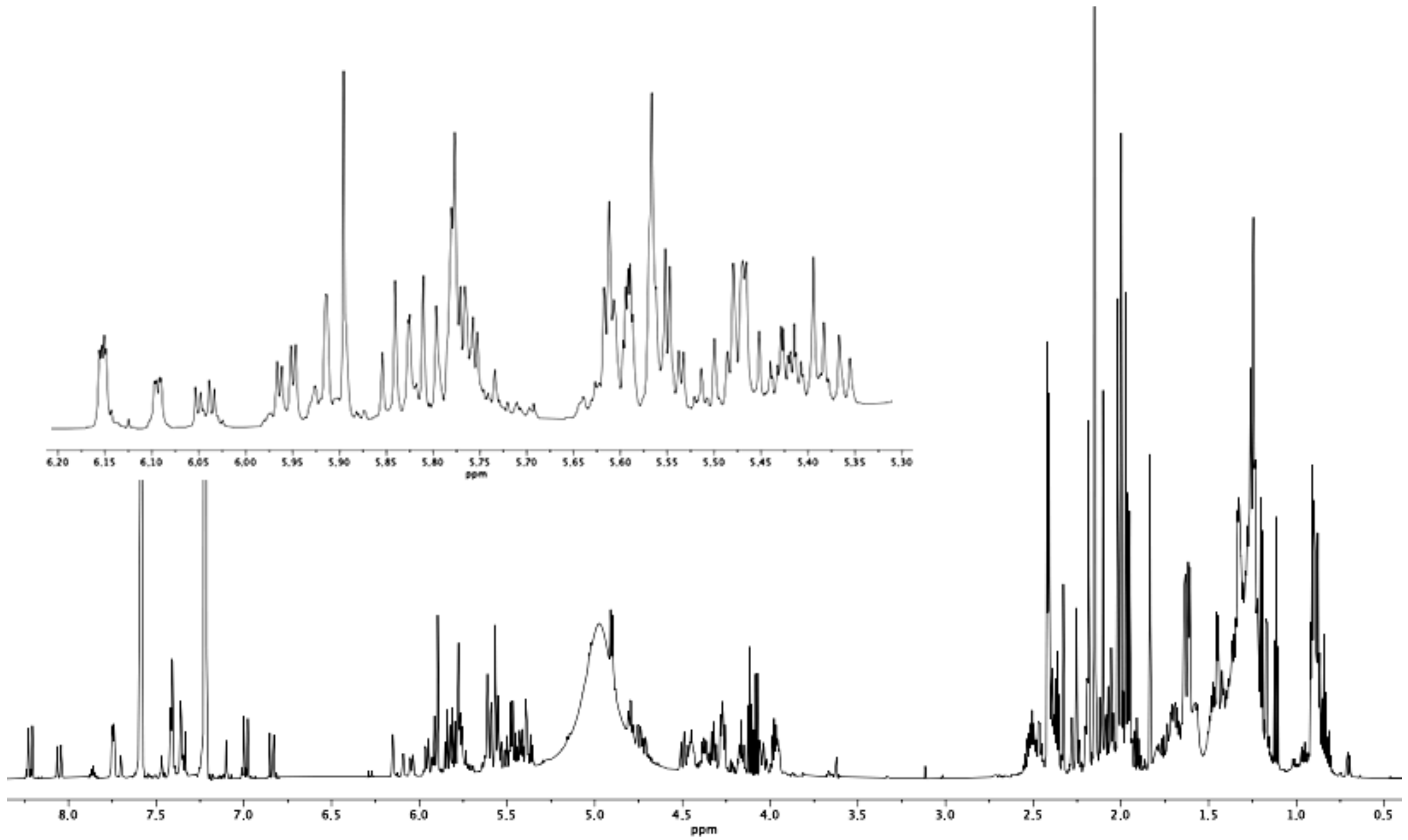

Figure S23.^{1}H-NMR spectrum (700 MHz) in pyridine-d_5 of the peracetylated mixture of compounds **7** and **8**.

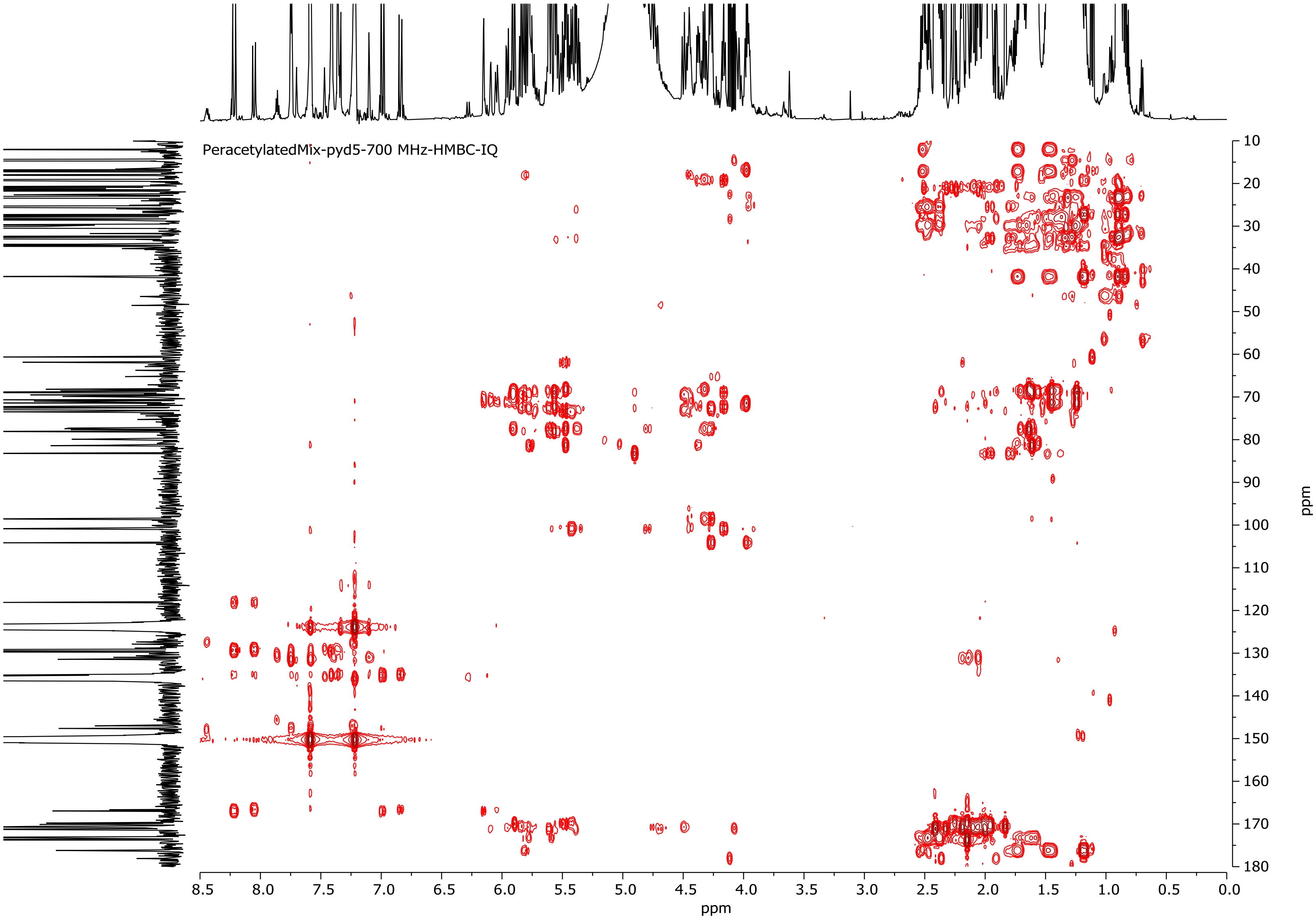

Figure S24. ^{1}H-Detected heteronuclear ($^{2,3}J_{CH}$) correlation (HMBC) spectrum in pyridine-d_5 of the peracetylated mixture of compounds **7** and **8** with high resolution 1D ^{1}H (700 MHz) and ^{13}C (175 MHz) projections.

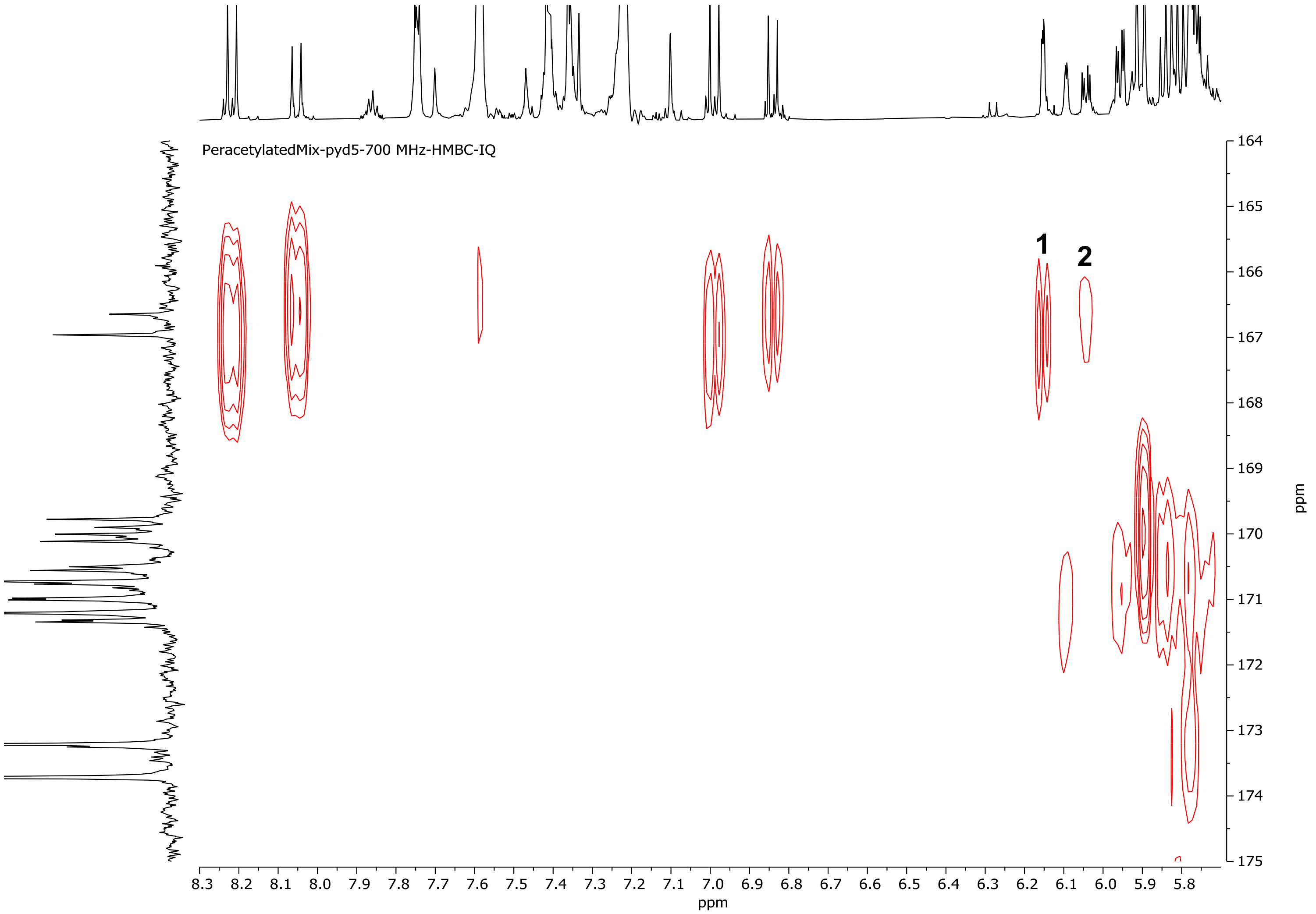

Figure S25. HMBC ($^{3}J_{CH}$) connectivity expansion for the esterification sequence in the peracetylated mixture for acetylated derivatives. Assignments for major compounds **7**: (**1**) Cna C-1/Rha″-2; and minor compound **8**: (**2**) Cna C-1/Rha″-3.

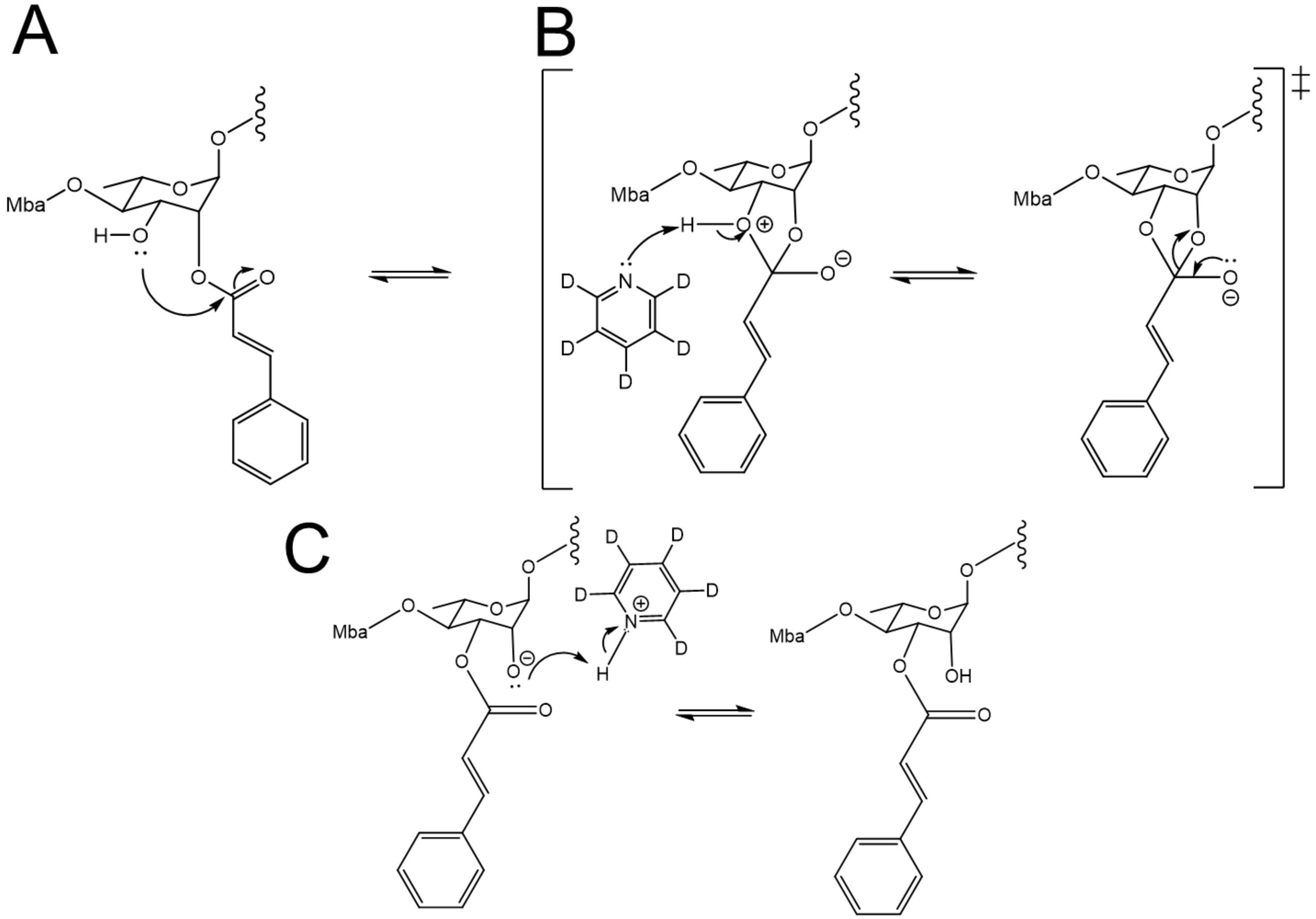

Figure S26. Proposed mechanism for the intramolecular transesterification between funisin I (**1**) and intrapilosin V (**3**). A) Nucleophilic attack by the neighboring –OH group promoting the axially oriented *O*-2 acyl migration; B) Transition state given by the formation of the *ortho*-ester intermediate and further base catalytic activity by pyridine; C) Final product of migration as the *O*-3 acyl form equatorially oriented.

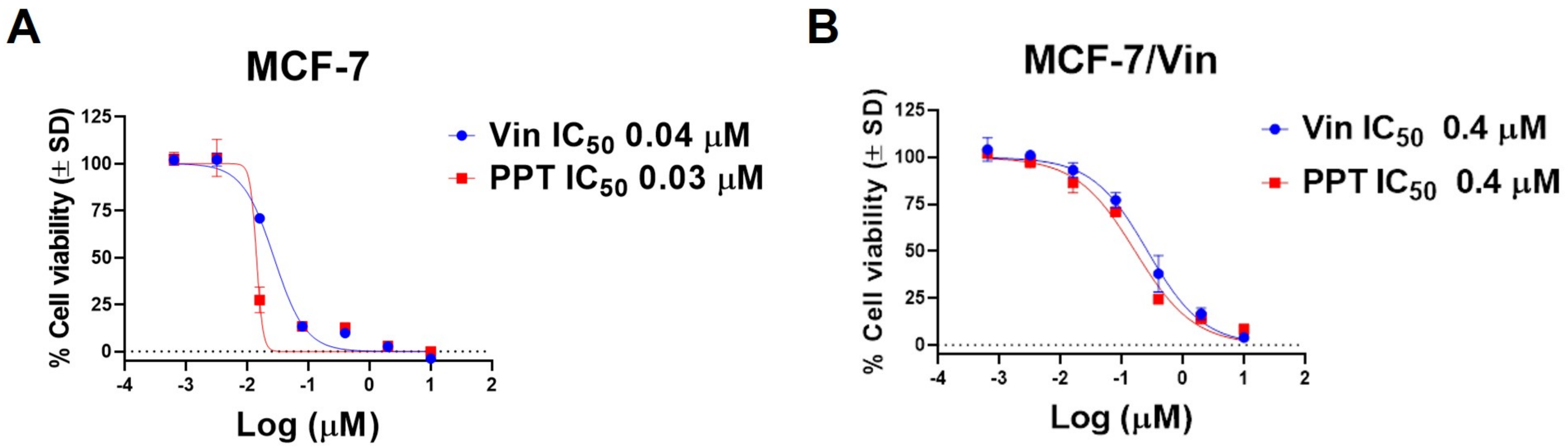

Fig. S27. Cytotoxicity of vinblastine and podophyllotoxin against MCF-7 and MCF-7/Vin after 72 h. Each experiment was performed three times independently (n =3). Results were expressed as mean ± standard deviation (SD).

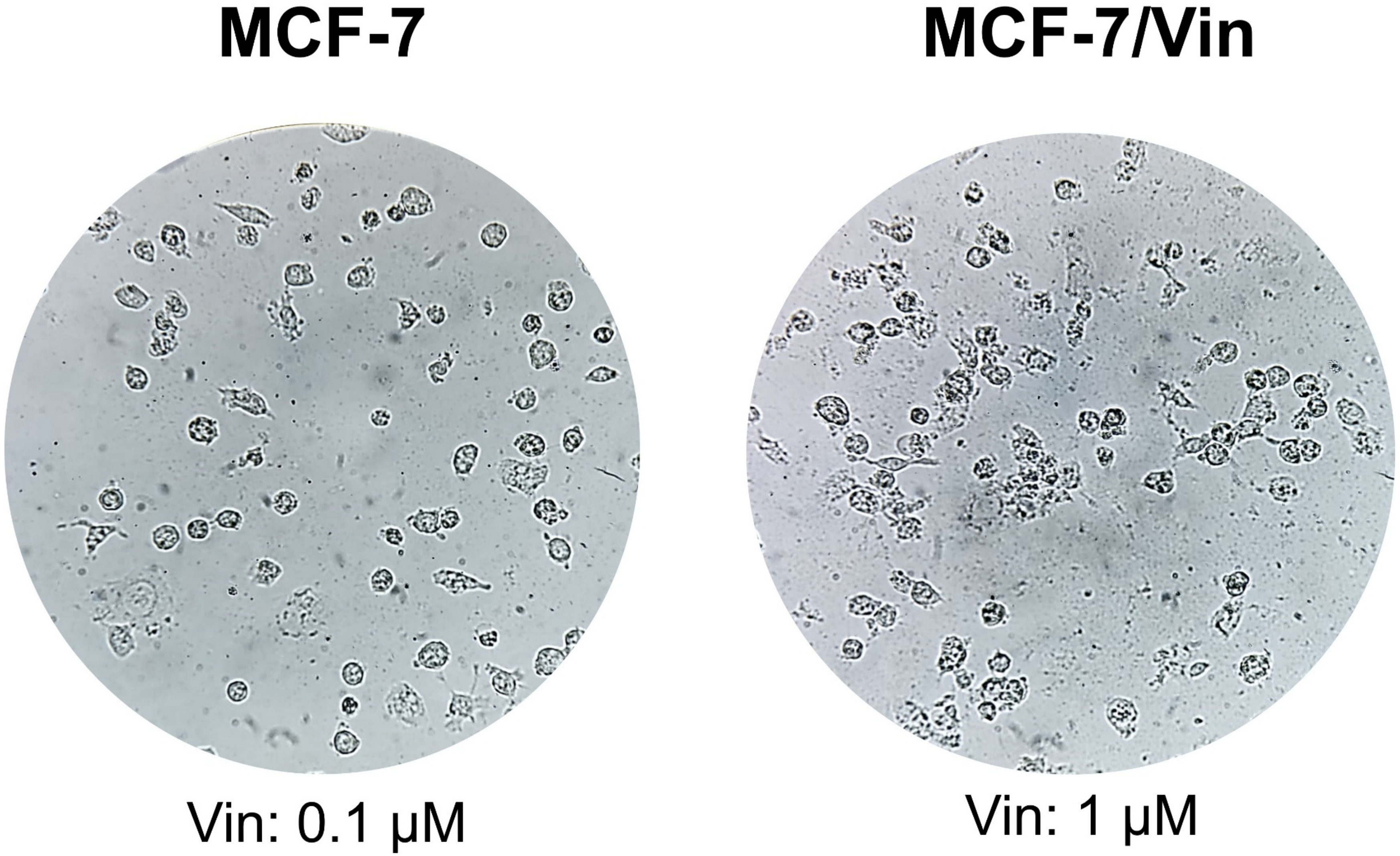

Figure S28. Images of vinblastine-sensitive MCF-7 cells and vinblastine-resistant MCF-7/Vin cells after 72 hours of treatment with vinblastine (Vin). Observation was performed under optical microscopy.

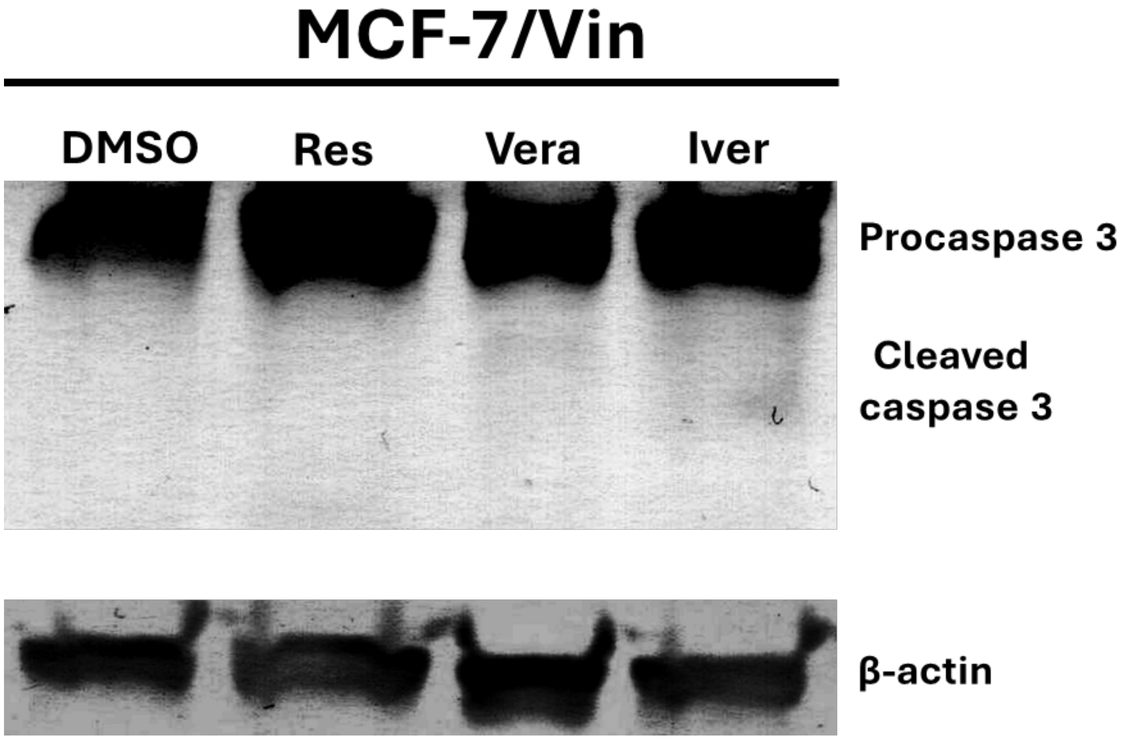

Figure S29. Activation of caspase-3 protein in MCF-7/Vin cells. Western blot analysis in vinblastine-resistant MCF-7 cells after 48 h of treatment with ABC pump inhibitors (Res: 10 µM; Vera: 10 µM, and Iver: 7 µM). DMSO: dimethylsulfoxide (control); Res: reserpine; Vera: verapamil; Iver: ivermectin.

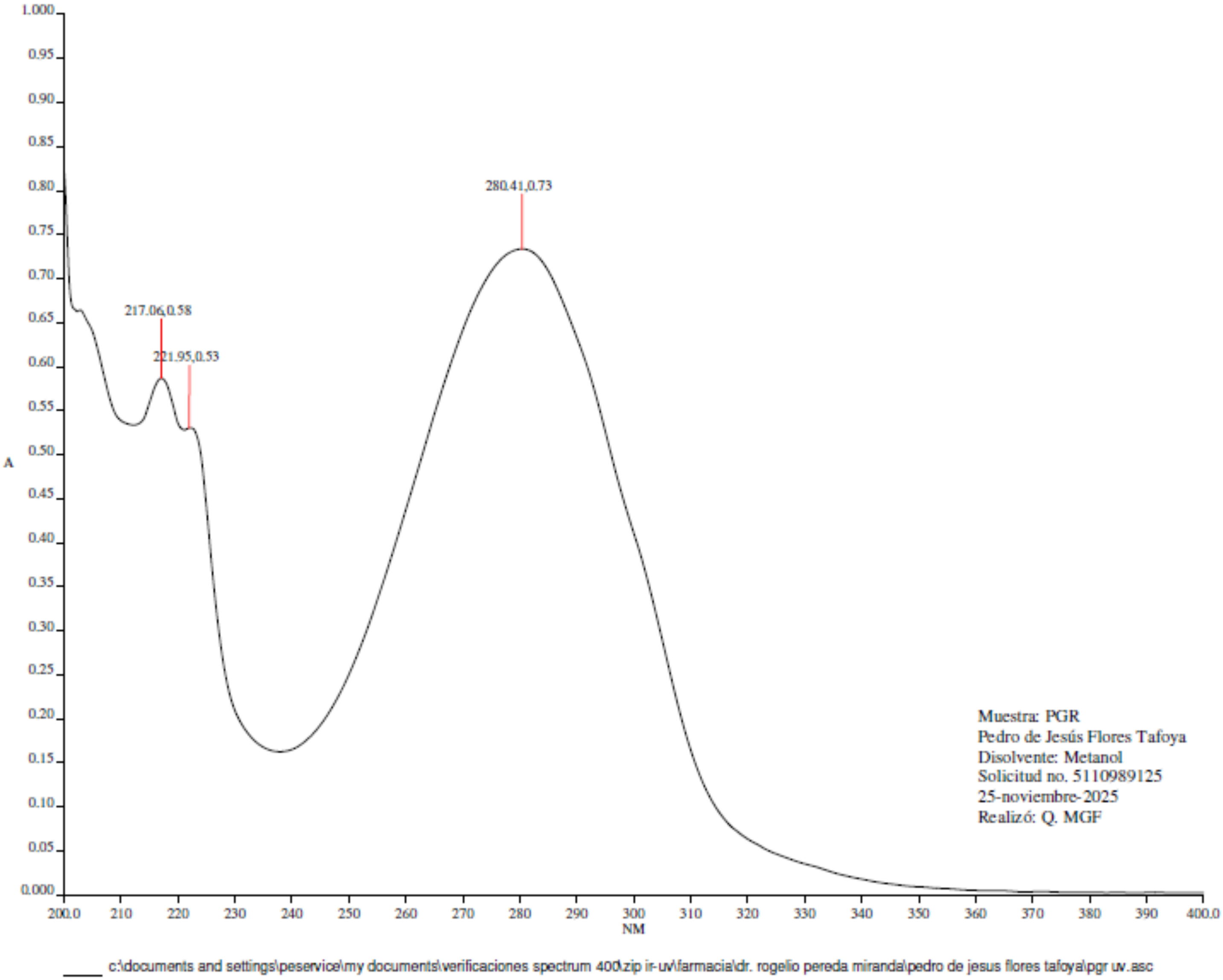

Figure S30. UV spectrum for funisin I (**1**)

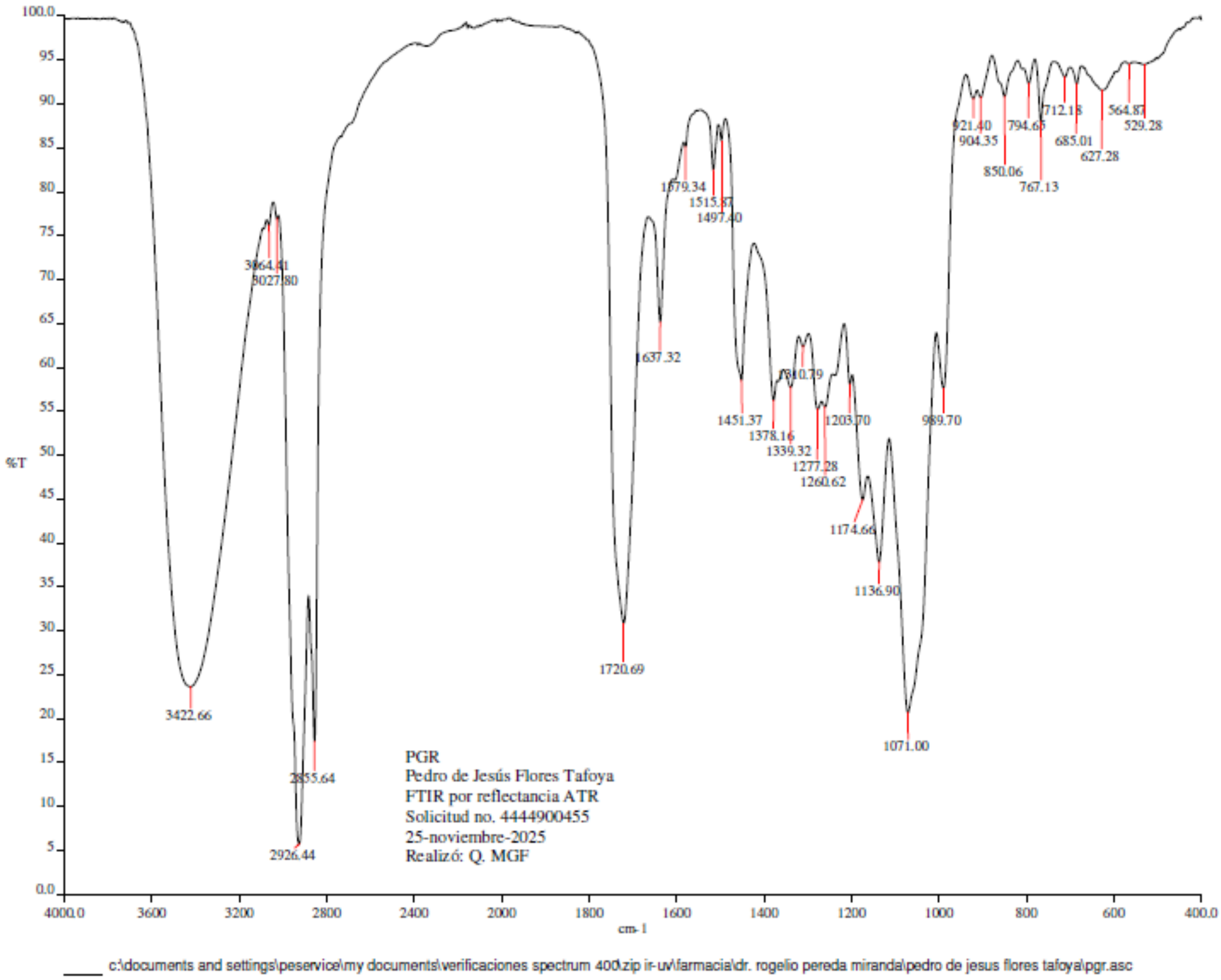

Figure S31. FTIR spectrum for funisin I **(1)**

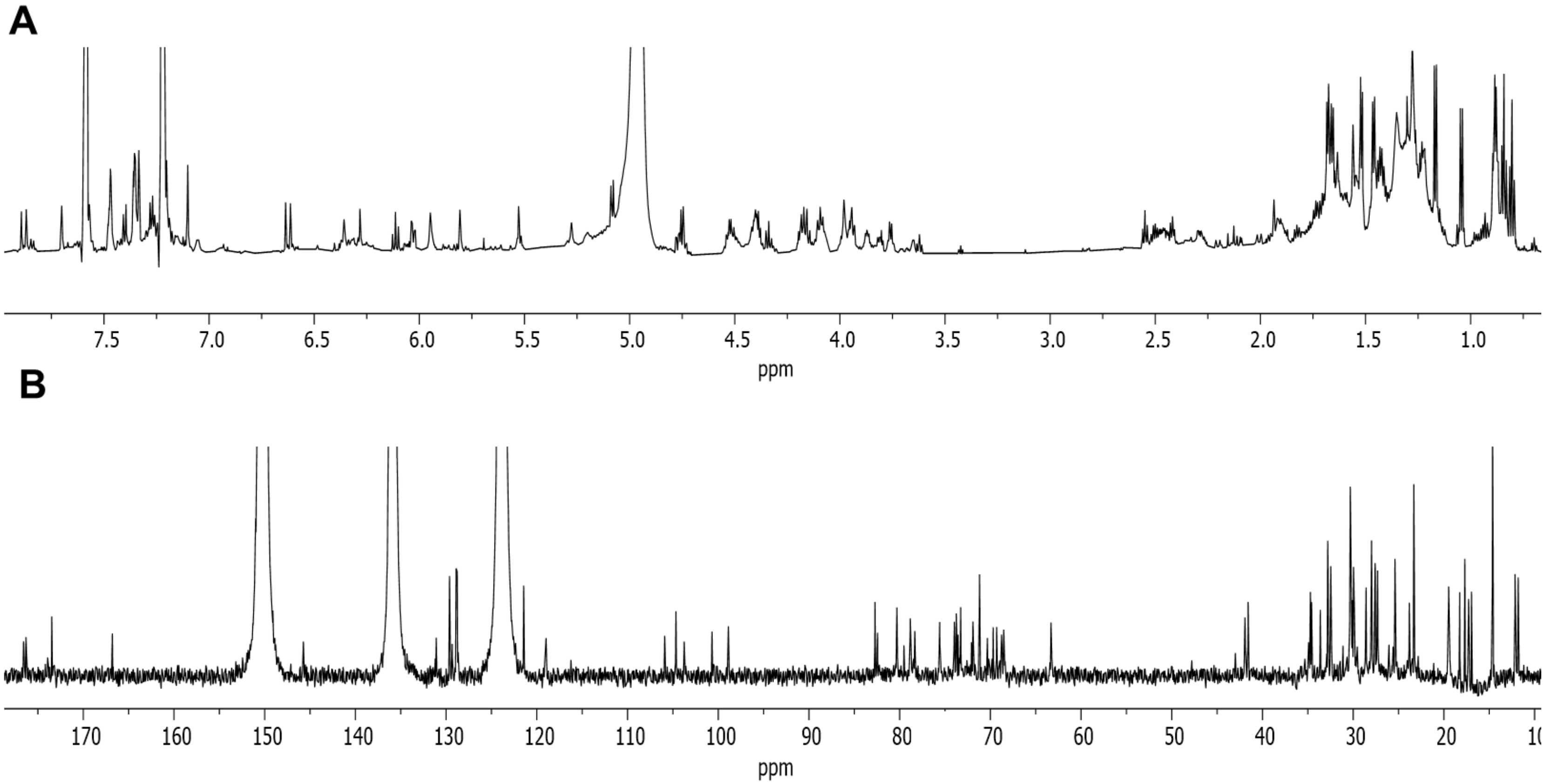

Figure S32. 1D-NMR spectra in pyridine-d_5 for intrapilosin I (2). ^{1}H (A, 700 MHz) and ^{13}C (B, 175 MHz)

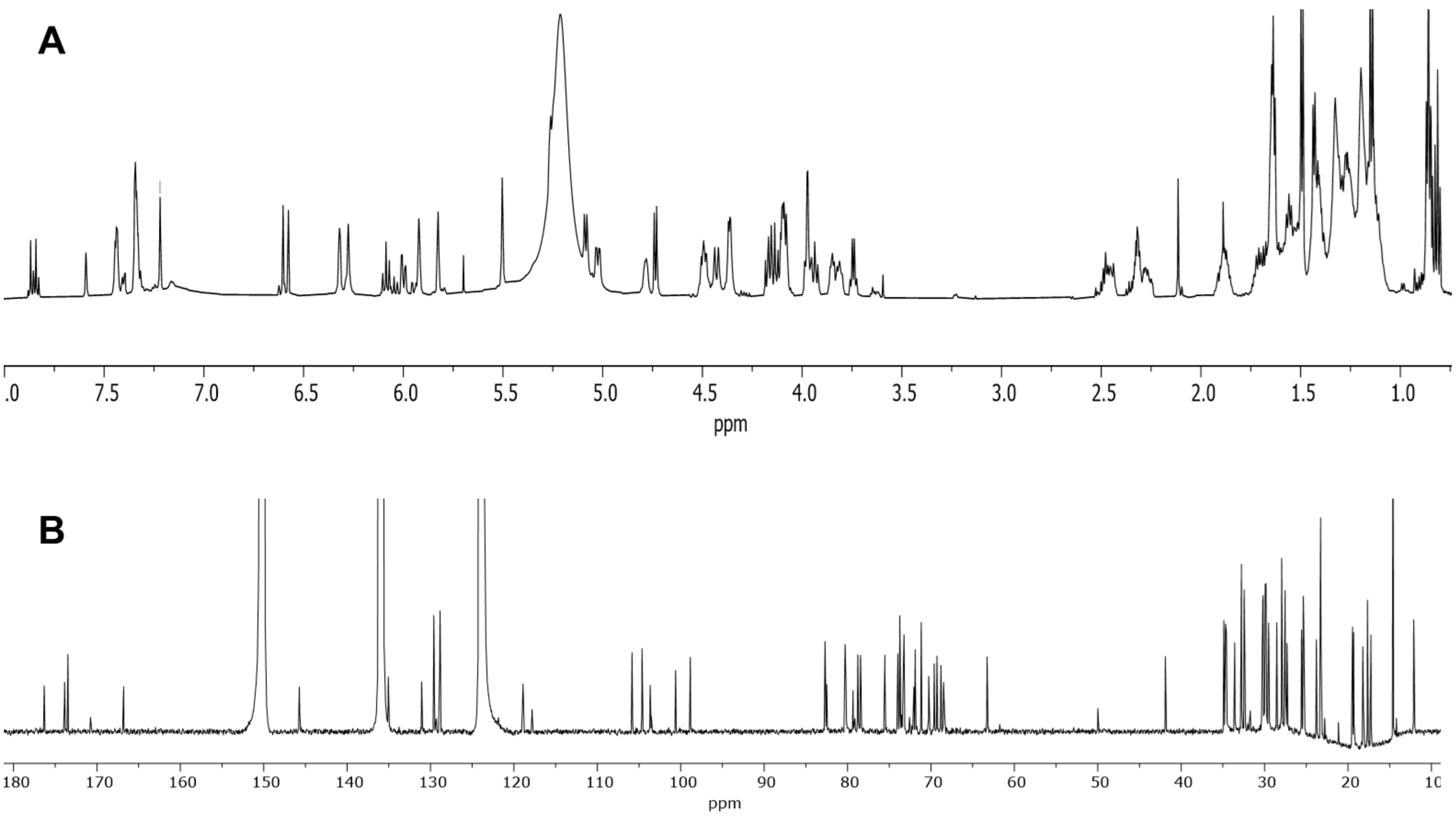

Figure S33. 1D-NMR spectra in pyridine-d_5 for intrapilosin V (3). ^{1}H (A, 700 MHz) and ^{13}C (B, 175 MHz)

Table S1. NMR Spectroscopic Data (700 MHz, pyridine-d_5) of compound **7**.

Position	δ_C, type	δ_H (*J* in Hz)
**Fuc-1**	104.1, CH	4.91 d (7.5)
**2**	68.2, CH	4.28 dd (9.4, 7.7)
**3**	72.7, CH	5.55 dd (9.4, 3.5)
**4**	73.5, CH	5.59 dt (3.5, 1.9)
**5**	77.5, CH	4.79 td (9.4 3.5)
**6**	19.3, CH_3	1.63 d (6.1)
**Rha-1**	100.8, CH	5.48 brs
**2**	71.1, CH	6.09 dd (3.5, 1.5)
**3**	69.8, CH	5.96 dd (10.3, 3.5)
**4**	77.6, CH	4.32 dd (9.2, 9.2)
**5**	77.5, CH	4.79 td (9.2, 3.5)
**6**	19.3, CH_3	1.64 d (6.3)
**Rha′-1**	98.5, CH	5.56 brs
**2**	70.6, CH	5.78 d (2.4)
**3**	71.0, CH	5.76 dd (9.5, 2.4)
**4**	81.4, CH	4.16 dd (9.5, 9.5)
**5**	68.8, CH	4.38 dq (9.5, 6.2)
**6**	19.1, CH_3	1.61 d (6.2)
**Rha″-1**	98.5, CH	5.91 brs
**2**	70.9, CH	6.15 dd (3.5, 1.7)
**3**	69.8, CH	5.96 dd (10.3, 3.5)
**4**	71.4, CH	5.81 t (9.8)
**5**	68.6, CH	4.45 dq (9.8, 6.6)
**6**	18.0, CH_3	1.46 d (6.1)
**Glc-1**	100.9, CH	5.38 d (7.9)
**2**	69.1, CH	5.50 dd (9.6, 9.6)
**3**	73.5, CH	5.84 dd (9.6, 9.6)
**4**	69.5, CH	5.46 dd (9.6)
**5**	69.5, CH	4.04 ddd (9.9, 2.8, 2.8)
**6a**		4.49 dd (13.0, 2.3)
**6b**	61.9, CH_2	4.75 dd (12.6, 3.5)
**Agl-1**	171.2, C	-
**2a**	34.7, CH_2	2.38 m
**2b**		2.50 m
**11**	83.2, CH	3.97 m
**16**	14.6, CH_3	0.90
**Dodeca-1**	173.2, C	-
**2**	34.7, CH_2	2.47 t (7.6)
**12**	14.6, CH_3	0.90
**2-mba-1**	176.2, C	-
**2**	41.8, CH	2.52 m
**2-Me**	17.2, CH_3	1.20 d (7.0)
**3**	25.5, CH_2	1.69 m
**4**	12.0, CH_3	0.91 (6.8)
**Cna-1**	166.9, C	-
**2**	118.1, CH	6.99 d (16.0)
**3**	147.6, CH	8.22 d (16.0)

Table S2. NMR Spectroscopic Data (700 MHz, pyridine-d_5) of compound **8**.

Position	δ_C, type	δ_H (J in Hz)
**Fuc-1**	104.1, CH	4.90 d (7.5)
**2**	68.2, CH	4.27 dd (9.8, 7.4)
**3**	72.8, CH	5.55 dd (9.8, 3.7)
**4**	73.5, CH	5.59 dt (3.7, 1.9)
**5**	77.5, CH	4.79 td (9.4 3.5)
**6**	19.3, CH_3	1.63 d (6.1)
**Rha-1**	100.8, CH	5.48 brs
**2**	71.1, CH	6.09 (3.5, 1.5)
**3**	69.8, CH	5.96 dd (10.3, 3.5)
**4**	77.6, CH	4.31 dd (9.5)
**5**	77.5, CH	4.79 td (9.4 3.5)
**6**	19.3, CH_3	1.64 d (6.3)
**Rha′-1**	98.5, CH	5.56 brs
**2**	70.6, CH	5.78 d (2.4)
**3**	71.0, CH	5.76 dd (9.5, 2.4)
**4**	81.3, CH	4.17 dd (9.5, 9.5)
**5**	68.8, CH	4.38 dq (9.5, 6.2)
**6**	19.1, CH_3	1.61 d (6.2)
**Rha″-1**	98.5, CH	5.91 brs
**2**	69.5, CH	5.47 m
**3**	69.9, CH	6.04 dd (10.3, 3.7)
**4**	71.4, CH	5.81 t (9.8)
**5**	68.6, CH	4.45 dq (9.8, 6.6)
**6**	18.0, CH_3	1.45 d (6.1)
**Glc-1**	100.7, CH	5.35 d (7.5)
**2**	69.1, CH	5.50 dd (9.6, 9.6)
**3**	73.5, CH	5.84 dd (9.6, 9.6)
**4**	69.5, CH	5.46 dd (9.6)
**5**	72.9, CH	4.04 ddd (9.9, 2.8, 2.8)
**6a**	61.9, CH_2	4.49 dd (13.0, 2.3)
**6b**		4.75 dd (12.6, 3.5)
**Agl-1**	171.2, C	-
**2a**	34.7, CH_2	2.38 m
**2b**		2.50 m
**11**	83.2, CH	3.97 m
**16**	14.6, CH_3	0.90
**Dodeca-1**	173.2, C	-
**2**	34.7, CH_2	2.36 t (7.6)
**12**	14.6, CH_3	0.90
**2-mba-1**	176.1, C	-
**2**	41.8, CH	2.52 m
**2-Me**	17.2, CH_3	1.17 d (7.0)
**3**	25.3, CH_2	1.38 m
**4**	12.1, CH_3	0.90 t (6.8)
**Cna-1**	166.3, C	-
**2**	118.1, CH	6.84 d (16.0)
**3**	147.0, CH	8.05 d (16.0)

Table S3. Cytotoxicity for isolated pure compounds (**1-3**) and control drugs in standard SRB assays.

Compound	IC_{50} (μM)	
	**MCF-7**	**MCF-7/ Vin**
**1**	>50	>50
**2**	>50	>50
**3**	>50	>50
Vin	0.04 ± 0.07	0.4 ± 0.06
PPT	0.03 ± 0.007	0.4 ± 0.006

Abbreviations: Vin, vinblastine; PPT, podophyllotoxin.

Table S4. NMR Spectroscopic Data (700 MHz, pyridine-d_5) of operculinic acid A (**6**).

Position	δ_C, type	δ_H (*J* in Hz)
**Fuc-1**	101.6, CH	4.81 d (7.8)
**2**	75.6, CH	4.52 dd (9.3, 7.8)
**3**	76.9, CH	4.17 dd (9.6, 3.5)
**4**	73.9, CH	3.95 d (3.5)
**5**	71.6, CH	3.82 q (6.4)
**6**	17.6, CH_3	1.54 d (6.4)
**Rha-1**	101.9, CH	6.25 d (1.5)
**2**	73.1, CH	4.69 dd (3.6, 1.6)
**3**	73.0 , CH	4.63 dd (9.5, 3.6)
**4**	82.6, CH	4.24 dd (9.5, 9.5)
**5**	67.9, CH	4.88 dq (9.5, 6.2)
**6**	19.1, CH_3	1.62 d (6.2)
**Rha′-1**	103.8, CH	5.91 s
**2**	72.1, CH	5.20 t (2.7)
**3**	83.1, CH	4.75 dd (9.1, 3.1)
**4**	78.9, CH	4.51 t (9.1, 9.1)
**5**	68.9, CH	4.41 dq (9.1, 6.2)
**6**	19.3, CH_3	1.61 d (6.2)
**Rha′-1**	103.5, CH	6.23 s
**2**	73.0, CH	4.90 dd (3.5, 1.6)
**3**	73.1, CH	4.45 dd (9.1, 3.5)
**4**	74.3, CH	4.24 dd (9.1, 9.1)
**5**	70.8, CH	4.32 dq (9.1, 6.2)
**6**	18.7, CH_3	1.59 d (6.2)
**Glc-1**	105.9, CH	5.25 d (7.5)
**2**	75.5, CH	4.00 dd (9.0, 7.5)
**3**	78.8, CH	4.22 dd (9.0, 9.0)
**4**	72.0, CH	4.13 dd (9.0, 9.0)
**5**	78.9, CH	3.99
**6a**		4.30 dd (11.9, 5.7)
**6b**	63.3, CH_2	4.55 dd (11.9, 2.6)
**Agl-1**	176.7, CH_2O	
**2a**		
	35.9, CH_2	2.53 t (7.5)
**2b**		
**11**	78.3, CH	3.99 m
**16**	14.2, CH_3	0.93 t
